# FMRP - G-quadruplex mRNA - miR-125a interactions: Implications for miR-125a mediated translation regulation of PSD-95 mRNA

**DOI:** 10.1371/journal.pone.0217275

**Published:** 2019-05-21

**Authors:** Brett DeMarco, Snezana Stefanovic, Allison Williams, Kathryn R. Moss, Bart R. Anderson, Gary J. Bassell, Mihaela Rita Mihailescu

**Affiliations:** 1 Department of Chemistry and Biochemistry, Duquesne University, Pittsburgh, Pennsylvania, United States of America; 2 Department of Cell Biology, Emory University School of Medicine, Atlanta, GA, United States of America; Korea University, REPUBLIC OF KOREA

## Abstract

Fragile X syndrome, the most common inherited form of intellectual disability, is caused by the CGG trinucleotide expansion in the 5’-untranslated region of the *Fmr1* gene on the X chromosome, which silences the expression of the fragile X mental retardation protein (FMRP). FMRP has been shown to bind to a G-rich region within the PSD-95 mRNA, which encodes for the postsynaptic density protein 95, and together with microRNA-125a to mediate the reversible inhibition of the PSD-95 mRNA translation in neurons. The miR-125a binding site within the PSD-95 mRNA 3’-untranslated region (UTR) is embedded in a G-rich region bound by FMRP, which we have previously demonstrated folds into two parallel G-quadruplex structures. The FMRP regulation of PSD-95 mRNA translation is complex, being mediated by its phosphorylation. While the requirement for FMRP in the regulation of PSD-95 mRNA translation is clearly established, the exact mechanism by which this is achieved is not known. In this study, we have shown that both unphosphorylated FMRP and its phosphomimic FMRP S500D bind to the PSD-95 mRNA G-quadruplexes with high affinity, whereas only FMRP S500D binds to miR-125a. These results point towards a mechanism by which, depending on its phosphorylation status, FMRP acts as a switch that potentially controls the stability of the complex formed by the miR-125a-guided RNA induced silencing complex (RISC) and PSD-95 mRNA.

## Introduction

Fragile X syndrome (FXS) is the most common form of inherited intellectual disability, being caused by the silencing of the fragile X mental retardation (*Fmr1*) gene, which encodes for the fragile X mental retardation protein (FMRP). FMRP is a messenger RNA (mRNA) binding protein whose role in translation control is essential for normal brain function [1]. FMRP has been proposed to function as a switch that regulates the local translation of neuronal mRNA targets in response to the cellular needs [2–4]. FMRP is part of a small family of RNA binding proteins, being most abundantly expressed in the brain and testes and characteristic in both fetal and adult tissues [5]. FMRP shares about 60% of amino acid identity with its autosomal paralogs, FXR1P and FXR2P [6, 7]. Even though it was proposed that these paralogs could partially compensate for the loss of FMRP, it was later shown that the expression of both FXR1P and FXR2P is not altered in patients with fragile X syndrome or in *Fmr1* knockout mice [8].

FMRP has a nuclear localization signal (NLS) and nuclear export signal (NES) which allows it to shuttle between the cytoplasm and nucleus [9]. FMRP associates with specific mRNAs in the nucleus in a sequence dependent manner, being recruited into ribonucleoprotein complexes, assisting in the transport of these mRNA targets to synaptic sites and regulating their translation in response to synaptic input [4]. FMRP has two types of RNA-binding motifs: three ribonucleoprotein K homology domains (KH0, KH1 and KH2) and an arginine-glycine-glycine (RGG) box [10]. The FMRP RGG box has been shown to recognize G-quadruplex structures of neuronal mRNA targets [11–15]. G-quadruplex structures are formed by G-rich sequences with four consecutive G-stretches leading to four guanine nucleotides assembling into a square planar arrangement, connected through Hoogsteen hydrogen bonding, stabilized by K^+^ ions [16, 17]. DNA G-quadruplexes require the presence of K^+^ ions for folding, while RNA G-quadruplexes of identical sequence can fold even in the absence of these ions, but have low stability [18]. The FMRP KH1 and KH2 domains have been shown to recognize the RNA structure called a kissing complex [19]. A kissing complex can form when two RNA hairpin loops interact with one another, typically through complementary base pairing from loop to loop and it has been shown that a stable kissing complex can with only two G-C loop-loop base pairs [20]. MicroRNA can have a kissing interaction with its mRNA target if the binding sequence is exposed in a hairpin loop leading to binding with minimal disruption to the mRNAs intramolecular base pairing and structure [21].

FMRP controls the production of the synaptic plasticity proteins in response of specific synaptic signals ([22] and references therein). It has been proposed that the FMRP phosphorylation plays a significant role in mediating the FMRP translation regulator function [3] allowing the balanced production of synaptic plasticity proteins. One important site of phosphorylation of FMRP is its highly conserved serine 500 residue in humans, through the action of the ribosomal S6 kinase, with the neuronal protein phosphatase 2A promoting the FMRP dephosphorylation [23, 24]. It has been shown that FMRP exists in both phosphorylated and unphosphorylated forms *in vivo*, with phosphorylated FMRP being predominantly present in dendritic granules [25, 26]. In response to metabotropic glutamate receptor (mGluR) activation, protein phosphatase 2A was shown to be a primary phosphatase that rapidly dephosphorylates FMRP in less than 30 seconds, with re-phosphorylation via ribosomal S6 kinase starting after 2 minutes [24]. It was further shown that phosphorylated FMRP inhibits the translation of its target mRNAs, while unphosphorylated FMRP allows translation to proceed [27].

**Fig 1:**
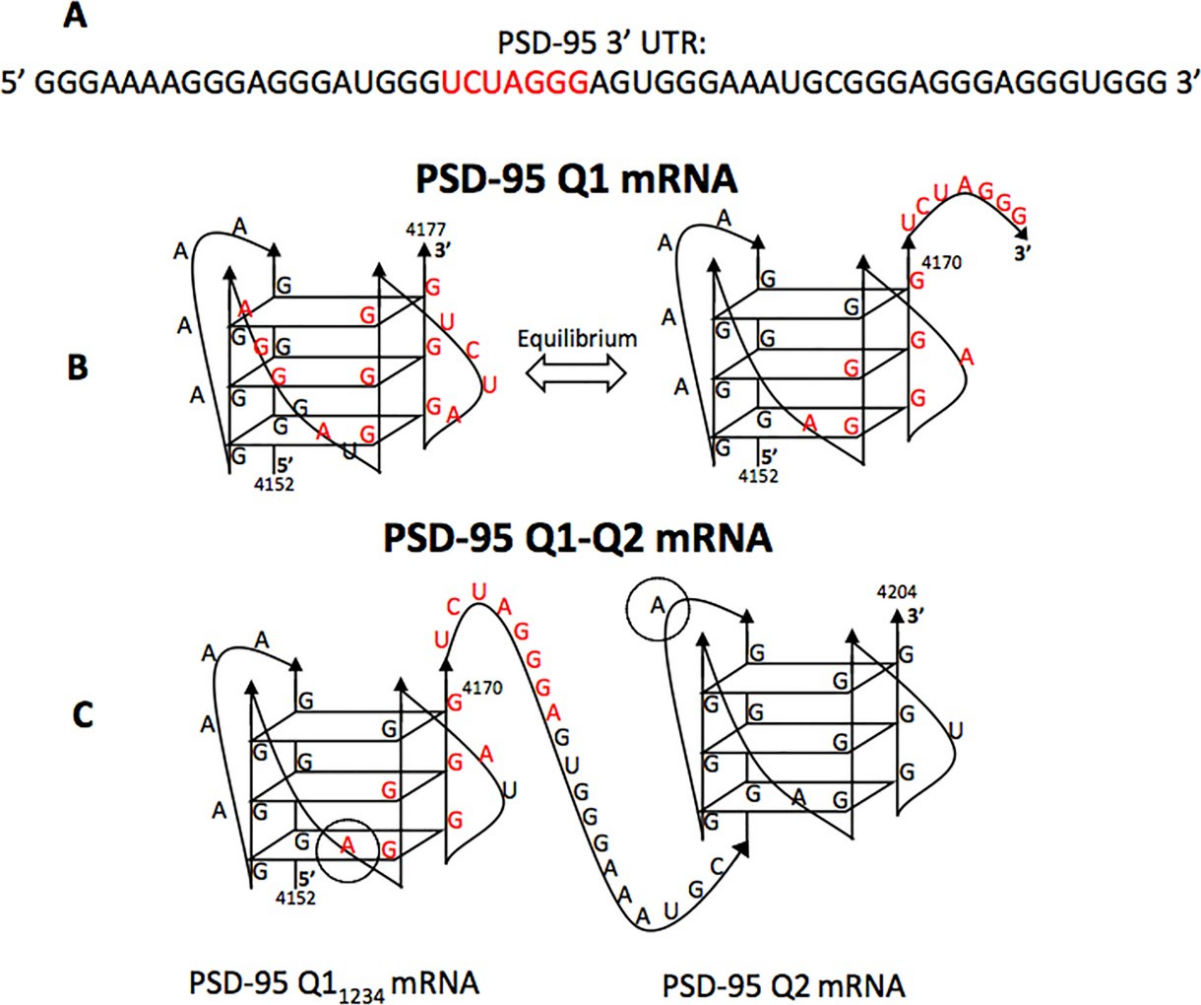
Proposed structures of the PSD-95 Q1 and Q2 G-quadruplexes. (**A**) PSD-95 3’ UTR sequence with the miR-125a seed binding site highlighted in red. **(B)** PSD-95 Q1 is shown in either the conformation that masks the miR-125a binding site (left) or the conformation Q1_1234_ that exposes it (right), respectively. **(C)** PSD-95 Q1-Q2 encompasses both the Q1 and Q2 G-quadruplexes. The miR-125a proposed binding site is shown in red, with the recognition site for its seed sequence being located in the linker between Q1_1234_ and Q2. Adenines 4162 and 4193 (circled in grey) were replaced with the fluorescent analog 2-amino purine (2-AP) for the fluorescence spectroscopy experiments in the isolated Q1_1234_ and Q2 G-quadruplexes, respectively.

The post synaptic density 95 (PSD-95) mRNA encodes for the PSD-95 protein which is part of the membrane-associated guanylate kinase family (MAGUK) of the postsynaptic scaffold proteins, and is essential for glutamate receptor localization and synapse function [28]. The PSD-95 mRNA translation is regulated by FMRP and miRNA-125a which work together to mediate the reversible inhibition of the PSD-95 mRNA translation in neurons [3]. Specifically, phosphorylated FMRP and the miR-125a-guided RISC act together to inhibit the PSD-95 mRNA translation, whereas upon FMRP dephosphorylation in response to synaptic triggers, the RISC complex dissociates from PSD-95 mRNA, allowing for the production of the PSD-95 protein [3]. FMRP has been shown to bind to a G-rich region within the 3’-UTR of PSD-95 mRNA [3] and in our previous study, we showed that this G-rich region which also encompasses the binding site for miR-125a adopts multiple G-quadruplex conformations some of which make accessible the miR-125a binding site (Fig 1) [29]. Here, we inquired if the role of FMRP in regulating PSD-95 mRNA translation is to remodel the mRNA structure, by binding to one or more G-quadruplexes in which the miR-125a binding site could be exposed/hidden, favoring the conformation in which the miR-125a site would be exposed. Our findings point towards a mechanism by which FMRP binds to the stable G-quadruplex structures within PSD-95 mRNA and depending on its phosphorylation status recruits the miR-125a-loaded RISC through its direct interactions with miR-125a and potentially with Ago2, contributing to the increased stability of the RISC-PSD-95 mRNA complex.

## Materials and methods

### RNA samples

PSD-95 Q1 [nt 4152–4177] (5’ GGGAAAAGGGAGGGAUGGGUCUAGGG 3’), PSD-95 Q1_1234_ [nt 4152–4170] (5’ GGGAAAAGGGAGGGAUGGG 3’), PSD-95 Q2 [nt 4190–4204] (5’ GGGAGGGAGGGUGGG 3’), PSD-95 Q1-Q2 [nt 4152–4204] (5’ GGGAAAAGGG AGGGAUGGGUCUAGGGAGUGGGAAAUGCGGGAGGGAGGGUGGG 3’) mRNAs were transcribed using synthetic DNA templates (TriLink BioTechnologies, Inc.) and expressed by T7 RNA polymerase driven *in vitro* transcription reactions. The RNAs were purified by 20% polyacrylamide, 8 M urea gel electrophoresis and electroelution and subsequently dialyzed against 10 mM cacodylic acid, pH 6.5 and their purity and migration according to difference in size was checked on a 20% denaturing gel (S1 Fig). miR-125a (5’ UCCCUGAG ACCCUUUAACCUGUGA 3’), miR-125a mutants (Mut: 5’ UCCCUGAGACCCUUUAGACU GUGA 3’ and Mut2 5’ UCCCUGAGACCGUUUAACCUGUGA 3’), miR-125b (5’ UCCC UGAGACCCUAACUUGUGA 3’), miR-122 (5’ UGGAGUGUGACAAUGGUGUUUG 3’), and miR-196a-1 (5’ UAGGUAGUUUCAUGUUGUUGGG 3’) were chemically synthesized by Dharmacon, Inc. Fluorescently labeled mRNAs, PSD-95 Q1_1234_ 11-2AP [nt 4157–4170] (5’ GGGAAAAGGG(2AP)GGGAUGGG 3’), PSD-95 Q2 4-2AP [nt 4190–4204] (5’ GGG(2AP)GGGAGGGUGGG 3’), and biotinylated PSD-95 Q1-Q2 (Bi PSD-95 Q1-Q2) were chemically synthesized by Dharmacon, Inc. All RNA sequences used in this study are listed in Table 1.

**Table 1:** RNA sequences used in this study.

Sequence Name	Nucleotide Sequence (5’ → 3’)
**PSD-95 Q1**	GGGAAAAGGGAGGGAUGGGUCUAGGG
**PSD-95 Q1**_**1234**_	GGGAAAAGGGAGGGAUGGG
**PSD-95 Q2**	GGGAGGGAGGGUGGG
**PSD-95 Q1-Q2**	GGGAAAAGGGAGGGAUGGGUCUAGGG AGUGGGAAAUGCGGGAGGGAGGGUGGG
**miR-125a**	UCCCUGAGACCCUUUAACCUGUGA
**miR-125a-Mut**	UCCCUGAGACCCUUUAGACUGUGA
**miR-125a-Mut2**	UCCCUGAGACCGUUUAACCUGUGA
**miR-125b**	UCCCUGAGACCCUAACUUGUGA
**miR-122**	UGGAGUGUGACAAUGGUGUUUG
**PSD-95 Q2 4-2AP Sub**	GGG(2AP)GGGAGGGUGGG
**PSD-95 Q1**_**1234**_ **2AP Sub**	GGGAAAAGGG(2AP)GGGAUGGG
**miR-196a-1**	UAGGUAGUUUCAUGUUGUUGGG

### FMRP RGG and HCV Peptide Synthesis

FMRP RGG peptide (RRGDGRRRGGGGRGQGGRGRGGGFKGNDDHSR) and HCV peptide (PRRGPRLGVRATRKTSERSQPRGRRQPIPKVRHQTGRRGSRPNWGPNDPRRRSRNLGK) were chemically synthetized and purified by the Peptide Synthesis Unit at the University of Pittsburgh, Center for Biotechnology & Bioengineering.

### FMRP expression and purification

FMRP Isoform 1 and its phosphomimic FMRP S500D [30, 31] were expressed and purified as described [32]. Briefly, the recombinant plasmids pET21a-FMRP encoding Isoform 1 (FMRP ISO1) fused with a C-terminal 6x histidine tag and pET21a-FMRP S500D were transformed in Rosetta 2(DE3) pLysS *E*. *coli* cells (Novagen). Single colonies were grown in 250 mL of LB + Ampicillin (Amp) + Chloramphenicol (Chl) at 37°C for 12 hours. Four sterile 2 L flask were prepared with 500 mL of LB + Amp + Chl and 25 mL of the cell culture was added to each flask, followed by incubation at 250 rpm at 37°C until an OD_600_ between 0.8–1.0 was reached. The expression of FMRP ISO1 or FMRP S500D was induced by adding 1 mM isopropyl β-D-1-thiogalactopyranoside (IPTG) to each flask, followed by incubation at 25°C for 12 hrs. The cells were then harvested, lysed and purified using Ni-NTA resin (Qiagen). The purified proteins were concentrated and dialyzed in a final buffer that contained 5% glycerol, 1 mM EDTA, and 300 mM LiCl. The concentration of the FMRP was determined by measuring absorbance at 280 nm using an extinction coefficient of 46370 M^-1^ cm^-1^.

### Native gel electrophoresis

Prior to their use in the native gels, the RNA samples were annealed by boiling for 5 minutes followed by incubation at room temperature for 10 minutes. KCl was added to the RNA samples at the desired concentration, prior to their annealing. For peptide binding investigation, FMRP RGG peptide was added to the annealed RNAs containing K^+^, allowing incubation at room temperature for 20 minutes. 20% native polyacrylamide gels in 0.5 X Tris/Borate/EDTA buffer and 5 mM KCl were run at 4°C, 88 V for 4.5 hours for PSD-95 Q1 and Q1_1234_ and for 4 hours for PSD-95 Q2. A 15% native polyacrylamide gel in 0.5 X Tris/Borate/EDTA buffer and 5 mM KCl was run at 4°C, 88 V for 4 hours for PSD-95 Q1-Q2. The gels were visualized by UV shadowing at 254 nm using an AlphaImager (AlphaInnotech, Inc.).

To observe the binding of miR-125a-Mut, miR-125a-Mut2, miR-125b, miR-122 and miR-196a-1 with FMRP ISO1 and FMRP S500D, the respective miRNA was incubated with the protein at room temperature for 15 min and then the samples were then run on 15% native gels with 0.5X Tris/Borate/EDTA buffer at 4°C and 88 V, for 2.5 h and visualized by SYBR Gold nucleic acid gel stain in an AlphaImager (AlphaInnotech, Inc.).

The effect of FMRP ISO1 or FMRP S500D upon miR-125a binding was also investigated. miR-125a, 200 nM, was annealed by boiling for 5 min, followed by incubation at room temperature for 10 min, after which FMRP ISO1 (or FMRP S500D), 400 nM, was added to the sample and incubated at room temperature for 15 min. 15% native gels were run with 0.5X Tris/Borate/EDTA buffer at 4°C and 88 V, for 2.5 h and visualized by SYBR Gold nucleic acid gel stain in an AlphaImager (AlphaInnotech, Inc.).

### Fluorescence spectroscopy

Steady-state fluorescence spectroscopy experiments were performed on a Horiba Jobin Yvon Fluoromax-3 fitted with a 150 W ozone-free xenon arc lamp. Experiments were performed in a 150 μL sample volume, 3 mm path-length quartz cuvette (Starna Cells). The excitation wavelength was set to 310 nm, the emission spectrum was recorded in the range of 330–450 nm, and the bandpass for excitation and emission monochromators were both set to 5 nm. FMRP ISO1 and FMRP S500D were titrated (50 nM) in the fixed concentration of PSD-95 Q1_1234_ 11-2AP or PSD-95 Q2 4-2AP (200 nM), monitoring the steady-state fluorescence changes as a result of protein-RNA interactions (each point was corrected for the fluorescence contributions originating from the protein). 1 μM of bovine serum albumin (BSA) was added in RNA sample prior to FMRP titration to prevent non-specific binding. The binding dissociation constant (Kd) was determined by fitting the binding curves to the equation:

$$F=1+\left(\frac{I_{B}}{I_{F}}-1\right)\frac{(K_{d}+{\left[P\right]}_{t}+{\left[RNA\right]}_{t})-\sqrt{{\left(K_{d}+{\left[P\right]}_{t}+{\left[RNA\right]}_{t}\right)}^{2}-4{\left[P\right]}_{t}{\left[RNA\right]}_{t}}}{2{\left[RNA\right]}_{t}}$$ (1)

where I_B_/I_F_ represents the ratio of the steady state fluorescence intensity of the bound and free mRNA, [RNA]_t_ is the total concentration of mRNA, and [P]_t_ is the total protein concentration. These experiments were performed in triplicate for each FMRP isoform, and the reported errors represent the standard deviations of the dissociation constants determined from independent fits to the three measurements.

The fluorescence spectra for FMRP ISO1 and FMRP S500D were acquired by setting the excitation wavelength at 295 nm to monitor the tryptophan fluorescence. The emission spectra were recorded in the range 310–400 nm and the bandpass for excitation and emission monochromators were both set to 5 nm. This was done for separate samples of each protein that had a final concentration of 1 μM and 5 μM, respectively in 10 mM cacodylic acid buffer, pH 6.5.

### Circular dichroism spectroscopy

All experiments were performed on a Jasco J-810 spectropolarimeter at 25°C, using a 1-mm path-length quartz cuvette (Starna Cells). 200 μL volumes of 1 μM and 5 μM FMRP ISO1 and FMRP S500D were prepared in 10 mM cacodylic acid buffer pH 6.5. The spectra were monitored from 180–350 nm and recorded by averaging a series of seven scans with a 1 sec response time and a 2 nm bandwidth. The spectra were corrected by subtracting the buffer contributions.

### RNA-based affinity pull-down assay

The biotinylated PSD-95 Q1-Q2 RNA probe in 10 mM cacodylic acid pH 6.5 containing 150 mM KCl was denatured at 95°C for 5 minutes and cooled at room temperature for 15 minutes. 5 μM probe was incubated for 20 minutes at room temperature with E17 mouse brain lysate obtained by lysing the cells using RIPA buffer (150 mM NaCl, 50 mM Tris-HCl, pH 8.0, 1% NP-40, 0.5% deoxycholate and 0.1% SDS) + Protease & RNase inhibitors. NeutrAvidin agarose (Thermo Scientific, Waltham, MA) pre-blocked with BSA was used to precipitate the probes. After extensive washing with RIPA buffer, 1/3 of the pellet was prepared for immunoblot and RNA was extracted from the other 2/3 of the pellet for quantitative real-time polymerase chain reaction (qPCR). Proteins were detected by immunoblot against FMRP (1:100, Clone 7G1-1, gift from Dr. Stephan Warren) and P-FMRP (1:1000, PhosphoSolutions, Aurora, CO).

### qPCR

Total RNA was recovered from input lysate and pull-down beads using TRIzol (Life Technologies). miRNA cDNA was prepared using qScript microRNA cDNA Synthesis Kit (Quanta Biosciences). qRT-PCR was performed on a Roche LightCycler 480 using SYBR Green (Roche) and the following primers: PerfeCTa Universal PCR Primer (Quanta), miR-124-3p UAAGGCACGCGGUGAAUGCC, miR-125a-5p UCCCUGAGACCCUUUAACCUGUG. Cp was calculated using the 2nd derivative method and relative concentrations were calculated from qPCR efficiency. For each biological replicate the pull-down concentrations were normalized to the miRNA level in the input sample. One-way ANOVA was performed using GraphPad Prism software. * = p-value < 0.05.

## Results

### Interactions of the FMRP RGG box with the G-quadruplexes formed within PSD-95 mRNA

It has been proposed that the FMRP phosphorylation plays a significant role in mediating its translation regulator function by binding to mRNA targets and allowing the balanced production of synaptic plasticity proteins [33]. FMRP has been shown to specifically bind to G-quadruplex structures formed within its mRNA targets through the involvement of its RGG box domain [11–15]. Muddashetty et al. demonstrated that the 3’UTR of PSD-95 mRNA contains specific binding sites for both FMRP and the microRNA miR-125a, that cooperate to regulate PSD-95 mRNA translation, which is dysregulated in neurons from *Fmr1* knockout mice [3]. The proposed model of the PSD-95 mRNA translation control suggests that FMRP remains bound to the mRNA in both its phosphorylated and unphosphorylated states, but the protein dephosphorylation in response to mGluR signaling triggers the miR-125a-guided RISC dissociation, allowing translation to occur [3].

**Fig 2:**
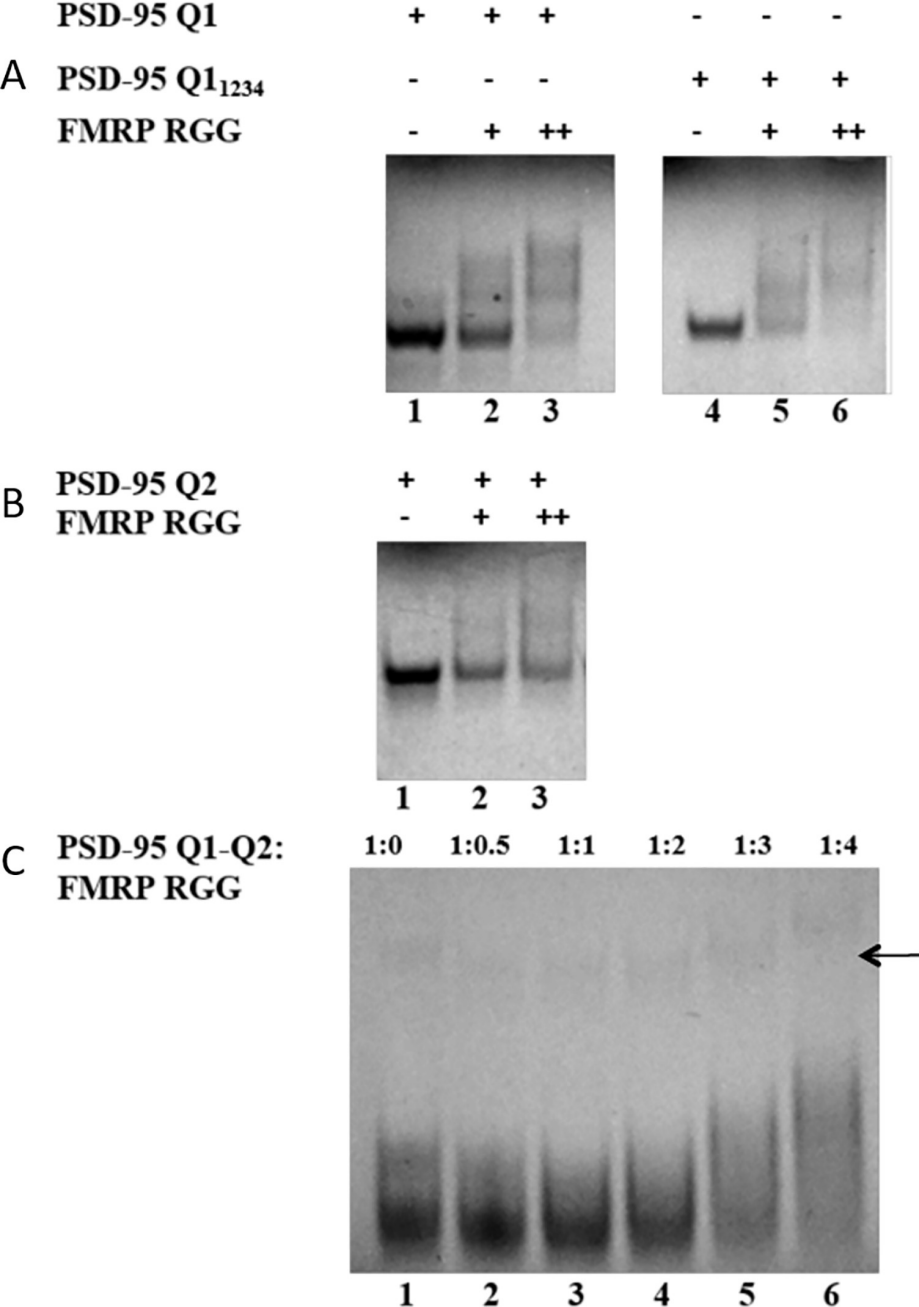
EMSA binding of FMRP RGG to the PSD-95 G-quadruplex structures. **(A)** EMSA (20% non-denaturing gel) of PSD-95 Q1 and PSD-95 Q1_1234_. Lane 1: free Q1; lanes 2 and 3: Q1 and 1:1 and 1:2 ratios of FMRP RGG; lane 4: free Q1_1234_; lanes 5 and 6: Q1_1234_ and 1:1 and 1:2 ratios of FMRP RGG **(B)** EMSA (20% non-denaturing gel) of PSD-95 Q2. lane 1: free Q2; lanes 2 and 3: Q2 and 1:1 and 1:2 ratios of FMRP RGG. **(C)** EMSA (15% non-denaturing gel) of PSD-95 Q1-Q2 with FMRP RGG. Lane 1: free Q1-Q2, lanes 2–6: Q1-Q2 and increasing ratios of FMRP RGG box from 1:0.5 to 1:4 as labeled at the top of the gel. 5 mM KCl was present in the RNA samples, gels and running buffer; the gels were visualized by UV shadowing at 254 nm. The arrow indicates dimeric stacked PSD-95 Q1-Q2 complexes [29].

In a previous study [29] we have shown that the PSD-95 3’-UTR G-rich region that contains the miR-125a binding site forms two G-quadruplex structures, named Q1 and Q2 (Fig 1B and 1C). PSD-95 Q1 [nucleotides (nt) 4152–4177] is highly dynamic being able to adopt multiple G-quadruplex conformations, only one of which exposes the miR-125a binding site (Fig 1A), whereas PSD-95 Q2 [nt 4190–4204] adopts a stable G-quadruplex structure (Fig 1B). Q1_1234_ [nt 4152–4170] is a truncated version of Q1 that lacks the last 7 nt, being locked in a single conformation. We hypothesized that FMRP binds the Q1, Q1_1234_ and Q2 G-quadruplexes, potentially playing a role in modulating the accessibility of the miR-125a to its binding site on PSD-95 mRNA. Given that the FMRP domain that binds G-quadruplex structures is its RGG box we initially analyzed the FMRP RGG box domain interactions with these G-quadruplexes by electromobility shift assays in 20% non-denaturing polyacrylamide gels in 0.5 X TBE buffer, using a chemically synthesized RGG peptide (FMRP RGG). The formation of the RNA-FMRP RGG complex was observed as the disappearance of the free RNA band (Fig 2A and 2B), as the peptide-RNA complex has an overall positive charge appearing blurry on the gel.

The Q1 G-quadruplex is dynamic and adopts multiple conformations [29]. This is evidenced by the presence of several bands one of which is dominant, in the free RNA (Fig 2A, lane 1), whereas a single band is present for the free Q1_1234_ and Q2 G-quadruplexes (Fig 2A and 2B lane 1). After their incubation at 1:1 and 1:2 RNA: peptide ratios, complexes form between the FMRP RGG and Q1 (Fig 2A, lanes 2, 3), Q1_1234_ (Fig 2A, lanes 5, 6) and Q2 (Fig 2B, lanes 1, 2) G-quadruplexes, as shown by the diminishing intensity of the free RNA bands, with the concomitant apparition of smeary bands at higher molecular weights.

To evaluate if FMRP RGG is still able to target these G-quadruplexes within an expanded Q1-Q2 segment which encompasses the Q1 and Q2 sequences and the linker between them [nt 4152–4204], we performed a similar EMSA experiment in 15% non-denaturing polyacrylamide gel in 0.5 X TBE buffer and 5 mM KCl (Fig 2C). We have previously shown that two conformations are present in the PSD-95 Q1-Q2 fragment in the presence of K^+^ ions, where both Q1 and Q2 G-quadruplexes are folded [29]. Two bands were observed for the free Q1-Q2 RNA (Fig 2C, lane 1) corresponding to these two alternative conformations [29], and upon addition of the FMRP RGG, both bands are shifted (Fig 2C, lanes 2–6), indicating the formation of a complex. Increasing ratios of FMRP RGG were used, as there are two G-quadruplex structures present on Q1-Q2. A very faint upper band indicated by the arrow in Fig 2C and corresponding to dimeric stacked PSD-95 Q1-Q2 molecules [29] is also present and this is also shifted in the presence of the FMRP RGG box. This result shows that the Q1 and Q2 G-quadruplexes within an expanded segment retain the ability to interact with FMRP RGG, as it was shown for the isolated Q1 and Q2 structures.

**Fig 3:**
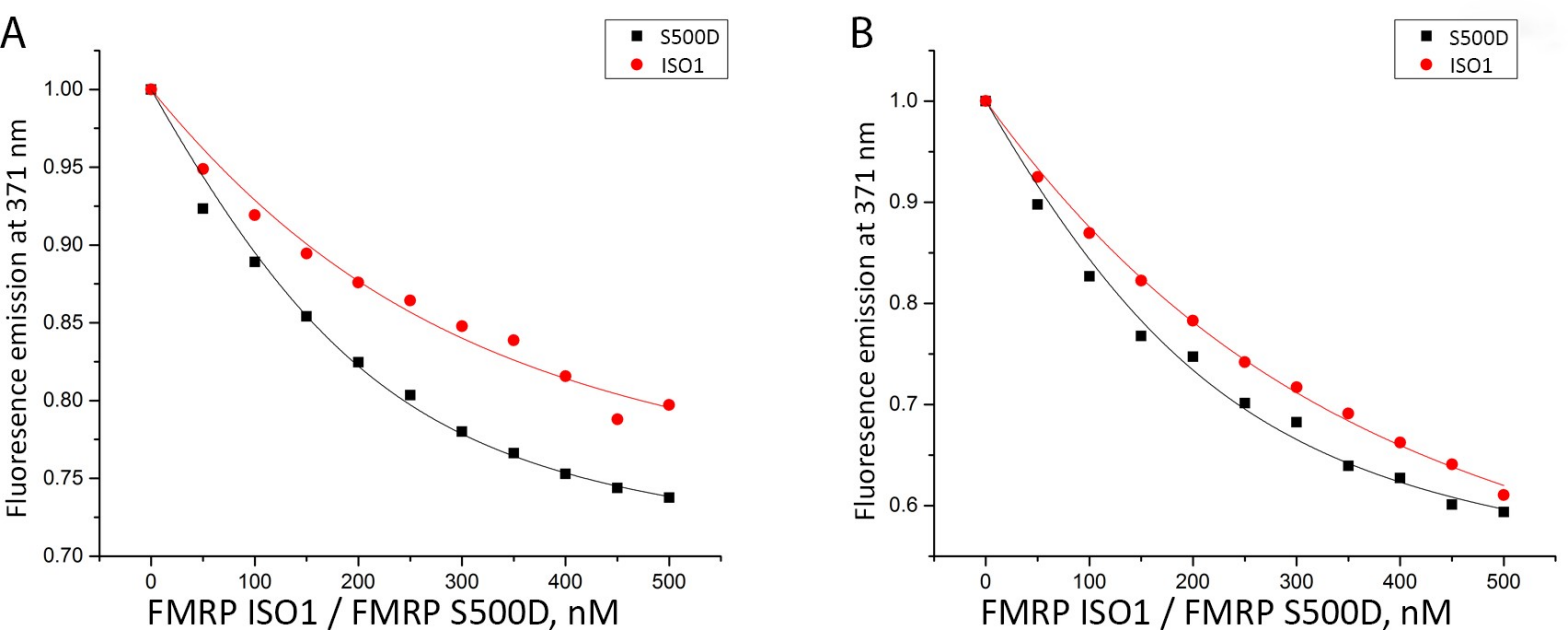
Fluorescence spectroscopy experiments measuring the binding of FMRP and FMRP S500D to PSD-95 Q1_1234_ and Q2 mRNAs. **(A)** Binding of PSD-95 Q1_1234_ 11-2AP by FMRP ISO1 (K_d_ = 128 ± 27 nM; shown by red circles), and FMRP S500D (K_d_ = 66 ± 10 nM; shown by black squares). **(B)** Binding of PSD-95 Q2 4-2AP by FMRP ISO1 (K_d_ = 195 ± 16 nM; shown by red circles) and by FMRP S500D (K_d_ = 100 ± 17 nM; shown by black squares).

We hypothesized that the role of FMRP is to remodel the PSD-95 mRNA structure by binding to one or more G-quadruplexes in which the miR-125a binding site could be exposed/hidden, regulating the RISC accessibility. Thus, it is possible that the phosphorylated FMRP targets with higher affinity a G-quadruplex in which the miR-125a binding site is exposed and available for binding. To test this hypothesis, we used fluorescence spectroscopy to measure the affinity of FMRP ISO1 and its phosphomimic FMRP S500D [31, 34] to the isolated Q1_1234_ and Q2 PSD-95 G-quadruplexes, respectively. We expressed and purified FMRP ISO1 and its phosphomimic FMRP S500D [32] and designed two fluorescently labeled RNAs, in which the adenines at position 4162 within PSD-95 Q1_1234_ and at position 4193 within PSD-95 Q2 were replaced with 2-aminopurine (2AP) (Fig 1C, adenines 4162 and 4193 are circled). 2AP is a highly fluorescent analog of adenine whose steady-state fluorescence is sensitive to changes in its microenvironment [35, 36]. Binding curves were obtained by titrating increments of FMRP ISO1 or FMRP S500D into a fixed 2-AP labeled RNA in 10 mM cacodylic acid buffer, pH 6.5 in 150 mM KCl (Fig 3). 1 μM of bovine serum albumin (BSA) was added to the RNA samples prior to FMRP titration to prevent non-specific binding.

The binding dissociation constants (K_d_) of the various FMRP-RNA complexes were determined by fitting the binding curves to Eq 1. All fluorescence spectroscopy experiments were performed in triplicate, and we report the average dissociation constant K_d_ and standard deviation. For both PSD-95 Q1_1234_ (Fig 3A) and Q2 (Fig 3B) mRNAs, it is apparent that the phosphomimic FMRP S500D targets G-quadruplexes with nearly 2-fold higher affinity (K_d_ PSD-95 Q1_1234_ = 66 ± 10 nM, K_d_ PSD-95 Q2 = 100 ± 17 nM), than FMRP ISO1 (K_d_ PSD-95 Q1_1234_ = 128 ± 27 nM, K_d_ PSD-95 Q2 = 195 ± 16 nM). It is interesting to note that the dephosphorylated FMRP still has high affinity for the Q1_1234_ and Q2 G-quadruplexes (albeit less than FMRP S500D), consistent with *in vivo* data showing that upon its dephosphorylation triggered by synaptic input, FMRP remains bound to PSD-95 mRNA [3].

**Fig 4:**
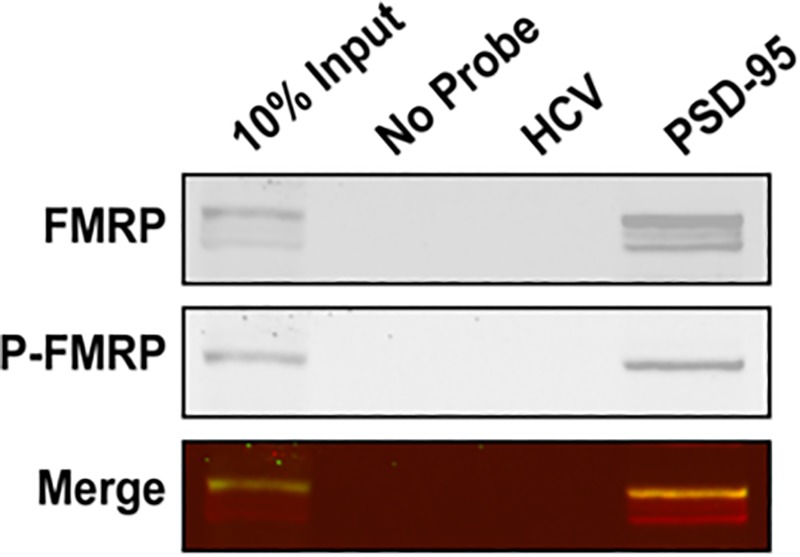
Bi-PSD-95 Q1-Q2 pull down of endogenous FMRP and phosphorylated FMRP. The Bi-PSD-95 Q1-Q2 probe was denatured at 95°C in 10 mM cacodylic acid pH 6.5 and 150 mM KCl for 5 minutes and cooled at room temperature for 15 minutes. 5 μM of probe was incubated with E17 mouse brain lysate for 20 minutes at room temperature and NeutrAvidin agarose (Thermo Scientific, Inc.) pre-blocked with BSA was used to precipitate the probes. After extensive washing, proteins were detected by immunoblot against FMRP (top panel) and its phosphorylated form P-FMRP (middle panel).

To further validate that the PSD-95 mRNA Q1-Q2 G-quadruplexes are sufficient for the endogenous FMRP recognition we have performed a series of pull-down experiments using a biotin-labeled PSD-95 Q1-Q2 probe (Bi-PSD-95 Q1-Q2). The probe was denatured at 95°C in 10 mM cacodylic acid pH 6.5 and 150 mM KCl for 5 minutes and cooled at room temperature for 15 minutes after which was incubated with lysates from E17 mouse brain in RIPA buffer and further precipitated with NeurAvidin agarose. Since the RIPA buffer contains 150 mM NaCl, we perfomed UV thermal denaturation experiments of the probe under the same conditions at 295 nm and showed that it retains the G-quadruplex fold as evidenced by the presence of two hypochromic transitions (S2 Fig). This result is consistent with our previous data showing that the Q2 G-quadruplex is very stable showing an incomplete hypochromic transition in as low as 25 mM KCl [29]. FMRP was identified using antibodies that recognize both non-phosphorylated and phosphorylated isoforms (Fig 4, top panel), while phosphorylated FMRP was identified using P-FMRP specific antibodies (Fig 4, middle panel). It is apparent that more than one FMRP isoform is pulled down from the brain lysate (shown as multiple bands in top panel), while only phosphorylated FMRP was recognized using P-FMRP specific antibody (shown as a single band in middle panel). In a control experiment, FMRP pull down was probed with a biotinylated HCV derived RNA probe [14, 29] for which FMRP has no specificity. These findings suggest that the G-quadruplexes within PSD-95 Q1-Q2 are sufficient for recognition by both FMRP and phosphorylated FMRP in the presence of other cellular factors, validating our *in vitro* fluorescence spectroscopy experiments.

### Interactions of FMRP with miR-125a

**Fig 5:**
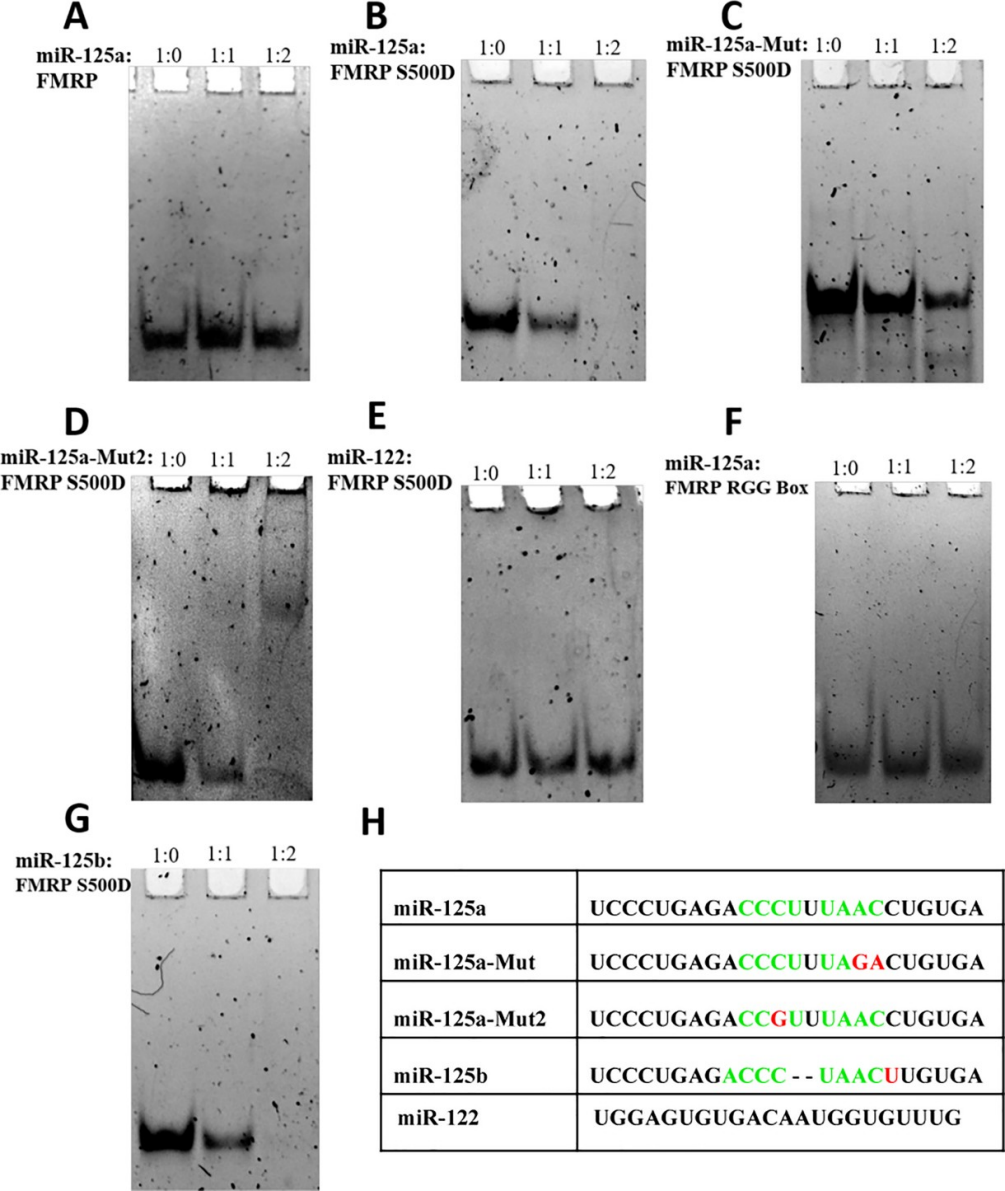
EMSA binding of FMRP ISO1, FMRP S500D binding to miR-125a. EMSA (15% non-denaturing gel) of miR-125a with FMRP ISO1 **(A)** and FMRP S500D **(B)**. Free 200 nM miR-125a (lane 1) was incubated with a 1:1 (lane 2) and 1:2 (lane 3) RNA: protein ratio. **(C)** and **(D)**. EMSA (15% non-denaturing gel) of two miR-125a mutants, miR-125a-Mut and miR-125a-Mut2, with FMRP S500D. Free 200 nM mutant miR-125a (lane 1) was incubated with a 1:1 (lane 2) and 1:2 (lane 3) RNA: FMRP S500D. **(E)**. EMSA (15% non-denaturing gel) of an unrelated miRNA, miR-122, with FMRP S500D. Free 200 nM miR-122 (lane 1) was incubated with a 1:1 (lane 2) and 1:2 (lane 3) RNA: FMRP S500D. **(F)**. EMSA (15% non-denaturing gel) of miR-125a with the FMRP RGG box. Free 200 nM miR-125a (lane 1) was incubated with a 1:1 (lane 2) and 1:2 (lane 3) RNA: FMRP RGG peptide ratio. **(G)** EMSA (15% non-denaturing gel) of miR-125b with FMRP S500D. Free 200 nM miR-125b (lane 1) was incubated with a 1:1 (lane 2) and 1:2 (lane 3) RNA: FMRP S500D ratio. The gels were visualized by staining with SYBR Gold for 15 minutes. The miRNA sequences used are shown in **(H)** with the predicted binding sites for the KH domains highlighted in green; miR-125a-Mut, miR-125a-Mut2, miR-125b, and miR-122 sequences are also shown for comparison with the differences from miR-125a highlighted in red.

It has been shown that recombinant FMRP binds miRNAs derived from Dicer cleavage and can anneal them onto their mRNA targets by using its KH1 and KH2 domains [37]. Thus, we investigated by EMSA if FMRP ISO1 and its phosphomimic FMRP S500D bind to miR-125a. Our results show that at 200 nM RNA concentrations the unphosphorylated FMRP does not bind miR-125a (Fig 5A), and that only FMRP S500D binds miR-125a (Fig 5B), as evidenced by the diminishing and respective disappearance of the free miR-125a band at 1:1 (lane 2) and 1:2 (lane 3) ratios of miRNA: protein. We have also demonstrated that the isolated FMRP RGG box domain does not bind miR-125a, indicating that this FMRP RNA binding domain it is not responsible for the recognition between FMRP S500D and miR-125a (Fig 5F). Additionally, the recognition of miR-125a is specific, as an unrelated miRNA miR-122, is not bound by FMRP S500D (Fig 5E).

The finding that at nM concentrations only the phosphomimic FMRP S500D binds miR-125a is novel, and apparently contradicts previous findings that unphosphorylated recombinant FMRP binds miRNAs [37, 38]. We have tested if FMRP ISO1 and FMRP S500D bind to miR-196a-1, one of the miRNAs used in a previous study [37], and found that at 200 nM RNA concentrations and with the protein added in ratios of 1:1 and 1:2 only FMRP S500D binds this miRNA and not FMRP ISO1 (S3 Fig). It is possible that in previous studies much higher ratios of FMRP to miRNA were used. The FMRP KH1 and KH2 domains are required for binding miRNA [37]. The KH RNA binding domain binds to single stranded RNA with the preferred four nucleotide sequence Y(C/A)(A/C)Y, where Y is a pyrimidine ([39, 40] and references therein). However, there are exceptions to this rule, as the first KH domain of the polyC binding protein 2 (PCBP2) has been crystalized in complex with both CCCT/U and ACCC sequences [41] and the third KH domain of the KSRP protein has been crystalized in complex with the AGGGU sequence [42]. We analyzed the sequence of miR-125a and determined that it has multiple sites that satisfy the Y(C/A)(A/C)Y sequence requirement: two overlapping sites within its seed (UCCC- nt 1–4 and CCCU- nt 2–5) and two additional adjacent sites (CCCU- nt 10–13 and UAAC nt 15–18) (Fig 5H). Typically, a single KH domain recognizes four nucleotides, however, tandem KH domains within the same protein have been shown to recognize longer RNA sequences [43–46]. The dissociation constants of single KH domains in complex with their RNA targets is in the low to high micromolar range, whereas those of full-length proteins containing tandem KH domains are in the low nM range [44, 47, 48].

Our EMSA binding experiments of FMRP S500D were performed at a fixed 200 nM miR-125a concentration and since the free miR-125a band disappeared completely in the presence of 400 nM FMRP S500D (Fig 5B, lane 3), we estimated that the dissociation constant of the FMRP S500D - miR-125a complex is in the low nM range. This result suggests that more than one KH domain could be involved in the FMRP S500D binding to miR-125a. Chmiel et al. have reported dissociation constants of 22 nM for the Drosophila P-element somatic inhibitor protein, that has three canonical and one non-canonical tandem KH domains, binding to an RNA target [44]. Mutations in one of the three canonical KH domains resulted in dissociation constants ranging from 0.1–1.3 μM, depending on which of the KH domains was mutated [44]. It has been shown that the individual KH domains of the KSRP protein bind to an RNA target with affinities ranging between 140 to more than 1000 μM [47], whereas the full-length protein containing four KH domains has a dissociation constant of 4 nM for the same RNA target [48]. Mutations introduced in each of the KH domains resulted in an increase of the dissociation constant between 2-fold to 13-fold [48].

FMRP has three KH domains, one non-canonical KH0 domain at its N terminus (amino acids 126–202) [49] and two canonical KH domains, the KH1 domain (amino acids 216–279) and KH2 domain (amino acids 281–425). The KH0 domain lacks the canonical GXXG sequence between the two alpha helices of the KH domain βααβ core and since it has been shown so far that KH domains lacking this sequence do not show independent nucleic acid binding capability [50], we ruled out its involvement in miR-125a binding. Both KH1 and KH2 domains have the GXXG sequence and moreover, it has been shown that they are required for the efficient FMRP annealing of miRNAs onto their targets [37]. Thus, we hypothesized that the two adjacent sites CCCU nt 10–13 and UAAC nt 15–18 within miR-125a are the target sites recognized by the KH1 and KH2 domains of FMRP S500D. To test this hypothesis, we rationalized that the elimination of one of the two sites will greatly reduce the FMRP S500D affinity for miR-125a. Thus, we mutated nucleotides 17 and 18 from AC to GA, changing the UAAC nt 15–18 site in wild type miR-125a to UAGA nt 15–18 in the mutated miR-125a (miR-125a-Mut), removing this site as a recognition motif for the KH domain (Fig 5H). As seen in Fig 5C, the affinity of FMRP S500D is greatly diminished for miR-125a-Mut as compared with the wild type miR-125a. We have also investigated the binding of FMRP S500D to miR-125b, a miRNA belonging to the miR-125 family, which has an identical sequence to miR-125a except it lacks nt 14 and 15 (UU) (Fig 5H). miR-125b will thus present to FMRP S500D either the CCCU nt 10–13 or the UAAC nt 13–16 (equivalent positions 15–18 in miR-125a) binding sites, but not both, since U13 is used in each of the two binding sites. Surprisingly, the binding affinity of FMRP for miR-125b seems to be very similar to that for miR-125a (compare Fig 5B and Fig 5G). It is possible that one of the binding sites remains UAAC nt 13–16, whereas in miR-125b the second binding site becomes ACCC nt 9–12. There is precedent for the recognition of the ACCC sequence by the KH domain, as this sequence has been crystalized in complex with the PCBP2 KH1 domain [51]. This result suggests that as long as the UAAC nt 13–16 site remains intact, sequence variations could be tolerated at the second binding site. To test this hypothesis in the context of miR-125a, we constructed miR-125a-mut2 in which the auxiliary binding site, CCCU nt 10–13, was mutated to CCGU, changing a single nucleotide from the preferred sequence Y(C/A)(A/C)Y, and leaving the UAAC nt 15–18 binding site intact. We observed that FMRP S500D binds miR-125a-mut2 similarly to the wild type miR-125a (compare Fig 5B and 5D) indicating that indeed sequence variations can be tolerated at the second binding site without affecting the overall binding affinity of FMRP S500D. Taken together, these results suggest that the UAAC site is the primary binding site for FMRP S500D. When the UAAC was disrupted in the miR-125a-Mut we saw a significant reduction in the binding (Fig 5C) but for each miRNA with the UAAC site intact (miR-125a, miR-125a-Mut2, miR-125b) the binding appeared to be relatively the same, with the full disappearance of the free miRNA band occurring at a 1:2 RNA: protein ratio.

While the UAAC site appears to be the primary binding site, a second site whose sequence can somewhat vary makes additional contributions to the binding, since FMRP S500D can still bind miR-125a, albeit with lower affinity, when the UAAC nt 15–18 is mutated (Fig 5C). The finding that the unphosphorylated FMRP does not bind miR-125a even at RNA: protein ratios up to 1:6 (S4 Fig), whereas FMRP S500D’s binding is complete at a RNA: protein ratio of 1:2 is novel. The only difference between FMRP and FMRP S500D is the replacement of serine at position 500 with aspartic acid, mimicking its phosphorylation, and S500 is not located within the KH1 or KH2 domains. It is possible that the protein dephosphorylation either induces major structural changes within its KH1 and KH2 domains, or induces a change in the arrangement of the FMRP domains with respect to each other that might block the ability of one or both its KH1 and KH2 domains to bind RNA.

**Fig 6:**
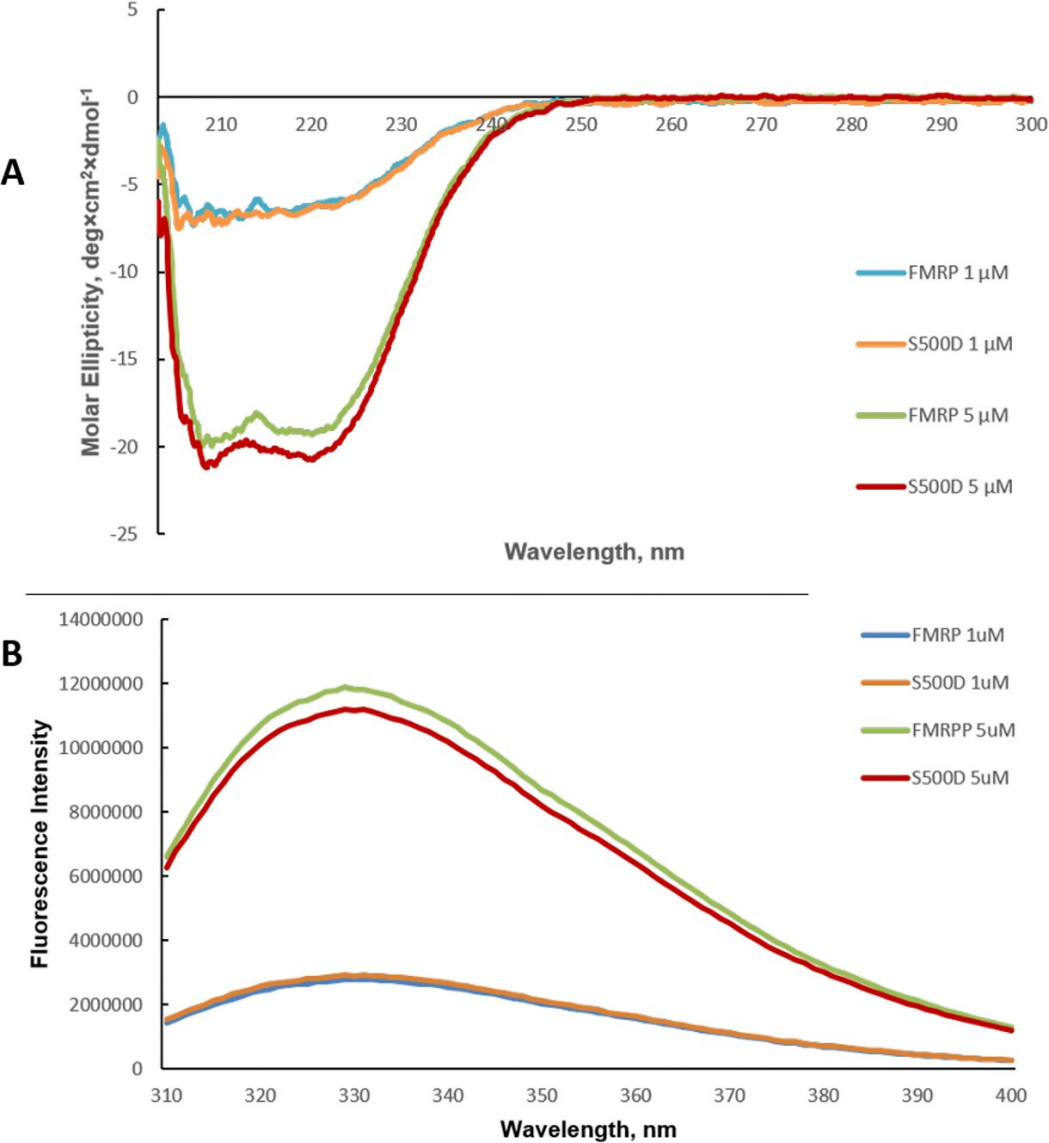
Biophysical characterization of FMRP and FMRP S500D. **(A)** CD spectra for FMRP and FMRP S500D were acquired at concentrations of 1 μM and 5 μM, respectively. **(B)** Fluorescence spectroscopy was used to monitor the tryptophan fluorescence of FMRP and FMRP S500D at 1 μM and 5 μM concentrations.

Thus, we investigated if the S500D mutation causes major structural changes within FMRP by circular dichroism spectroscopy and by fluorescence spectroscopy. We used FMRP and FMRP S500D at 1 μM and 5 μM concentrations, as it has been proposed that at high μM concentrations FMRP tends to aggregate [52]. As seen in Fig 6A there are no major structural differences between FMRP and FMRP S500D, as both their CD spectra overlap almost perfectly at both protein concentrations investigated.

In the fluorescence spectroscopy experiments we monitored the intrinsic fluorescence of the FMRP tryptophan residues, the observed fluorescence signal being an average value of the five tryptophan amino acids present in FMRP: W36, W79, W80, W395, W513. The first three tryptophans are located in the FMRP N terminal domain that contains two tandem Agenet 1 and Agenet 2 domains and its KH0 domain [49]. W395 is located in the KH2 domain variable loop, whereas W513 is located 13 amino acids from the S500 phosphorylation site and 19 amino acids from position 532, the beginning of the RGG box, most likely in an unstructured region. As seen in Fig 6B, the average tryptophan fluorescence spectra of FMRP and FMRP S500D are almost identical, consistent with the absence of major structural changes of the protein upon its dephosphorylation.

The crystal structure of the linked FMRP KH1 and KH2 domains reveals that there is no extensive contact between the domains, which are connected by a single amino acid, Glu280 [53]. It is possible that the two domains bind RNA independently of each other, and subtle protein conformation changes induced by phosphorylation could affect one domain or both. The S500 position is in the close proximity of the FMRP RGG box that binds G-quadruplex structures and our results show that although FMRP and FMRP S500D have an identical RGG box, they have different affinities for the Q_1234_ and Q2 G-quadruplexes (Fig 3). Thus, we speculate that the FMRP phosphorylation might affect the arrangement of the RGG box with respect to the KH1 and/or KH2 domains, in a conformation that allows both, the RGG box to bind with higher affinity to G-quadruplex structures and the KH1 and KH2 domains to both bind miR-125a. Since the FMRP RGG box is unstructured in the absence of RNA, such a conformational change will not necessarily be apparent in CD spectral differences between FMRP and FMRP S500D. Moreover, since both W395 and W513 are located in unstructured regions, they might also not be able to report on the arrangements of the FMRP domains with respect to each other. Further work is required to elucidate how the FMRP phosphorylation allows the protein to bind with high affinity to both miR-125a and the PSD-95 G-quadruplex structures, whereas upon its dephosphorylation its affinity for miR-125a is greatly dimished whereas that for the G-quadruplex structures is reduced almost by half.

### miR-125a binding to PSD-95 mRNA in the presence of FMRP and FMRP S500D

**Fig 7:**
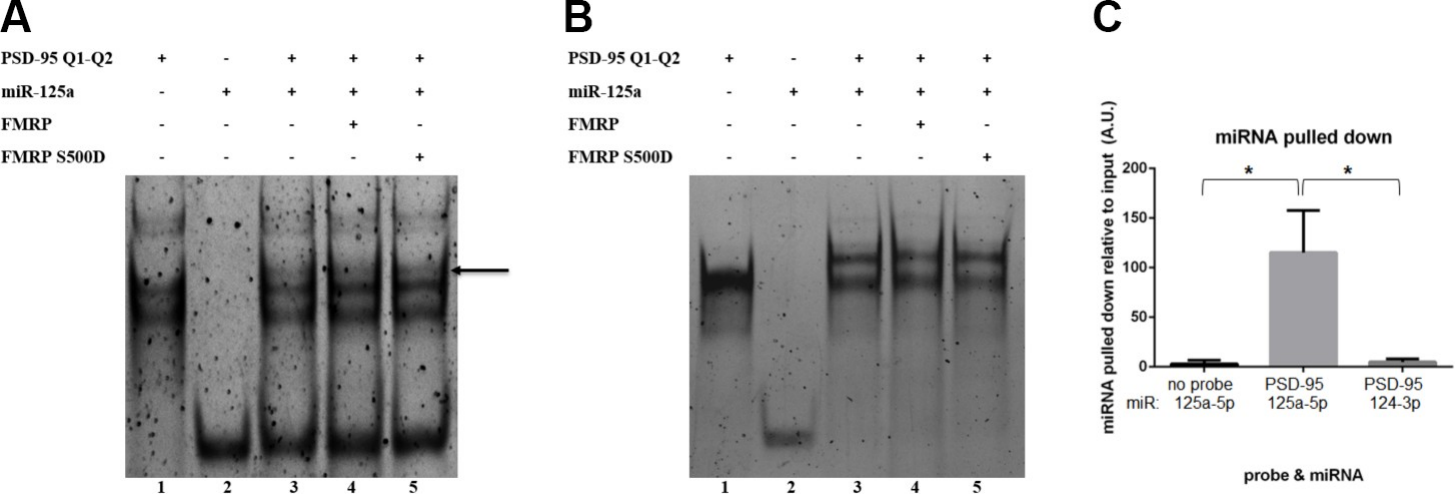
EMSA binding of PSD-95 Q1-Q2 and miR-125a in the presence of FMRP. **(A)** EMSA (15% non-denaturing gel) of PSD-95 Q1-Q2 mRNA with miR-125a in 25 mM KCl. 200 nM mRNA and miR-125a (lanes 1 and 2) were incubated together in the absence (lane 3) or presence of 400 nM FMRP or FMRP S500D (lanes 4 and 5). Following this incubation, proteinase K was added to digest FMRP and FMRP S500D prior to running the samples on the gel. **(B)** Identical conditions as described in (A) were used, except in the presence of 25 mM LiCl. **(C)** miR-125a was detected by qPCR experiments, in pull-down experiments from E17 mouse brain lysates by using the Bi-PSD-95 Q1-Q2 probe. A control miRNA, miR-134-3p, was not significantly pulled down by the Bi-PSD-95 Q1-Q2 probe.

In our previous study [29] we have shown that at micromolar RNA concentrations miR-125a binds to its target within PSD-95 mRNA, without the disruption of the G-quadruplex structures formed in Q1-Q2 and that the FMRP RGG box domain peptide did not affect this binding. However, the full-length FMRP contains the additional KH1 and KH2 RNA binding domains that we postulated bind to miRNA-125a and thus, might play a role in the binding of miR-125a to its target sequence within PSD-95 mRNA. To assess such a role we performed EMSA binding experiments in conditions less optimal for binding, at nanomolar RNA concentrations and reduced incubation time. The binding of miR-125a to PSD-95 Q1-Q2 was tested in the absence and presence of FMRP ISO1and FMRP S500D (Fig 7) in two different conditions: in 25 mM KCl and in 25 mM LiCl. The K^+^ ions stabilize G-quadruplex structures and we have shown previously [29] that in 25 mM KCl both Q1 and Q2 quadruplexes are folded. In contrast, we determined by UV thermal denaturation that in 25 mM LiCl the Q1 G-quadruplex is unfolded, whereas the Q2 G-quadruplex remains folded (S5 Fig). Thus, in 25 mM LiCl the miR-125a binding site on PSD-95 Q1-Q2 is fully exposed, whereas in 25 mM KCl Q1-Q2 exists in two alternate conformations both of which have the Q1 and Q2 G-quadruplexes folded, making the miR-125a seed binding site exposed in the linker between Q1 and Q2, but the rest of the binding site inaccessible [29]. In 25 mM KCl at 200 nM RNA concentration PSD-95 Q1-Q2 exists in two conformations as evidenced by two distinct main bands (Fig 7A, lane 1). Additionally, two very faint upper bands are present in lane 1, that we attribute to the stacking of two PSD-95 Q1-Q2 molecules, stabilized by K^+^ ions [29]. Upon the incubation of PSD-95 Q1-Q2 with miR-125a for 15 minutes in the absence of FMRP, a very faint band appears at a higher molecular weight (Fig 7A, lane 3, arrow), indicating the formation of the miR-125a-PSD-95 Q1-Q2 complex; however, most of the PSD-95 Q1-Q2 and miR-125a remain unbound. To test if FMRP ISO1 or FMRP S500D have chaperone activity with respect to the binding of miR-125a on PSD-95 Q1-Q2 [37], we have incubated them in the presence of the respective protein for 15 minutes, followed by treatment with proteinase K to digest FMRP ISO1 or FMRP S500D, prior to running the samples on the gel. The band corresponding to the miR-125a-PSD-95 Q1-Q2 complex has the same intensity in the absence or presence of the respective protein (compare Fig 7A, lanes 3, 4 and 5), indicating that neither FMRP ISO1 (lane 4), nor FMRP S500D (lane 5) have nucleic acid chaperone activity, as they do not stably anneal miR-125a upon its PSD-95 Q1-Q2 target. This result is in contrast to a previous report showing that FMRP has the ability to anneal miRNAs upon their RNA targets [37]; however, in that case the miRNA target site was not embedded into a G-quadruplex forming region.

To determine if the folding of the Q1 quadruplex has any effect upon the FMRP nucleic chaperone activity, we repeated the above experiments in the presence of 25 mM LiCl. In 25 mM LiCl at 200 nM RNA concentration PSD-95 Q1-Q2 exists mainly in a single conformation (Fig 7B, lane 1), whereas upon its incubation with miR-125a for 15 minutes in the absence of FMRP (Fig 7B, lane 3) a complex is formed as evidenced by the apparition of a shifted upper band with the concomitant complete disappearance of the free miR-125a band (compare Fig 7B, lanes 2 and 3). Moreover, a thin band is visible at a much higher molecular weight, which we attribute to the binding of two miR-125a molecules to PSD-95 Q1-Q2, as when the Q1 G-quadruplex is unfolded there are two binding sites complementary to the miR-125a seed sequence. Similarly, we have incubated miR-125a on PSD-95 Q1-Q2 in the presence of FMRP ISO1 or FMRP S500D for 15 minutes, followed by treatment with proteinase K to digest the proteins. FMRP ISO1 (Fig 7B, lane 4) or FMRP S500D (Fig 7B, lane 5) does not make any difference with respect to the formation of the miR-125a-PSD-95 Q1-Q2 complex, however, such an effect would be hard to detect in these conditions, given that even in the absence of the protein, miR-125a binds so efficiently to PSD-95 Q1-Q2 when the Q1 G-quadruplex is unfolded.

Our experiments do not support the hypothesis that FMRP and/or FMRP S500D have nucleic acid chaperone activity with respect to the binding of miR-125a to PSD-95 Q1-Q2, as in such a case the miR-125a-PSD-95 Q1-Q2 complex should be stable even after the protein digestion by proteinase K. This *in vitro* experimental design does not allow us to directly evaluate the FMRP ISO1 and FMRP S500D effects upon the formation of the miR-125a-PSD-95 Q1-Q2 complex, only to evaluate its nucleic acid chaperone activity.

To test if miR-125a binds to the PSD-95 Q1-Q2 RNA probe in the presence of endogenous FMRP we performed real-time quantitative polymerase chain reaction (qPCR) on the pull-downs using the Bi-PSD-95 Q1-Q2 probe with the E17 mouse brain lysates from Fig 4. As seen in Fig 7C middle bar, the Bi-PSD-95 Q1-Q2 probe which pulled down FMRP and FMRP S500D (Fig 4) also showed a significant pull down of miR-125a from the brain lysates in comparison to a control miRNA, miR-124-3p. However, since the probe pulled down both FMRP and FMRP S500D, we cannot conclude from these experiments that the pulled-down miR-125a was present only in the complexes containing FMRP S500D. These results show only that the miR-125a complementary sequence within PSD-95 Q1-Q2 is available for binding in the presence of endogenous FMRP, suggesting that the favored G-quadruplex conformation within the probe is one in which the miR-125a site is exposed.

## Discussion

In this study we have shown that both FMRP ISO1 and its phosphomimic, FMRP S500D bind to the PSD-95 Q1 and Q2 G-quadruplexes with high affinity and specificity *in vitro*, with the phosphomimic targeting both structures with greater affinity than the unphosphorylated protein. The dissociation constants of FMRP S500D, K_d_ PSD-95 Q1_1234_ = 66 ± 10 nM, K_d_ PSD-95 Q2 = 100 ± 17 nM), and FMRP ISO1 (K_d_ PSD-95 Q1_1234_ = 128 ± 27 nM, K_d_ PSD-95 Q2 = 195 ± 16 nM, are the first reported binding values of FMRP to PSD-95 mRNA. We have previously reported that FMRP binds with high affinity to G-quadruplex structures formed within Shank1 mRNA [14], NR2B mRNA [15] and its own encoding *Fmr1* mRNA, additional targets of the protein [54] Interestingly, for NR2B mRNA and one of the Shank1 mRNA G-quadruplexes a similar trend was observed where the affinity of FMRP S500D was higher, whereas there was no difference in affinity for the binding of FMRP ISO1 and FMRP S500D for its own mRNA. This difference might be related to the function of FMRP with respect to the particular mRNA, as FMRP is involved in the translation regulation of PSD-95, Shank1 and NR2B mRNAs, whereas in the case of *Fmr1* mRNA, FMRP is involved in its alternative splicing to affect the production of the isoforms ISO1, ISO2 and ISO3 [55]. Additionally, we have shown that the isolated PSD-95 Q1-Q2 G-quadruplexes are sufficient for the recognition of various isoforms of FMRP found within mouse brain lysates, including the phosphorylated FMRP (P-FMRP) (Fig 4), in agreement with our *in vitro* findings that both the unphosphorylated and phosphorylated FMRP target the G-quadruplex structures. Moreover, the structures adopted by these G-quadruplexes allow miR-125a to recognize its binding site, both *in vitro* and in mouse brain lysates (Fig 7).

Interestingly, we found that the FMRP-phosphomimic S500D binds to miR-125a (Fig 5B), whereas the unphosphoryated protein does not (Fig 5A), even at high protein: RNA concentrations (S4 Fig). This is a novel finding, as previously it has been shown that phosphorylated FMRP associates with precursor microRNAs [56, 57], and that FMRP can anneal mature miRNAs onto their mRNA targets [37], but not that depending on its phosphorylation status FMRP could function as a switch in binding mature miRNAs. The binding of miR-125a is specific, as an unrelated miRNA, miR-122, is not bound by FMRP S500D (Fig 5E). The FMRP RGG box is not involved in miR-125a binding (Fig 5F) and we proposed that FMRP S500D uses its KH1 and KH2 domains to bind the adjacent CCCU and UAAC sites within miR-125a which are canonical recognition sites for the KH domain RNA binding motif. A mutant miR-125a in which the UAAC site is disrupted, is still bound by FMRP S500D, albeit with much lower affinity (Fig 5C), supporting the idea that the protein binds multiple sites on miR-125a. While our data supports the proposal that FMRP S500D uses its KH1 and KH2 domains to bind miR-125a, it does not directly proves these interactions. Direct evidence can only be obtained by testing the binding of FMRP S500D containing mutations that abolish their KH domains’ RNA binding ability and such studies are part of an ongoing investigation in our laboratory.

Given that the FMRP phosphorylation confers its ability to bind miR-125a, we investigated if this posttranslational modification induces major secondary structure changes of the protein. Thus, we have used CD spectroscopy and fluorescence spectroscopy to compare for the first time the spectra of full-length FMRP and FMRP S500D, our results indicating that there are no major structural changes between FMRP and its phosphomimic (Fig 6). Based on our findings, we postulate that the FMRP phosphorylation induces rearrangements of both the KH1 and KH2 domains and of its RGG box with respect to each other that allow the RGG box to bind with higher affinity the PSD-95 Q1 and Q2 structures (Fig 3), and position the KH domains in the right orientation to optimally bind miR-125a (Fig 5).

There is evidence in the literature showing a link between FMRP and the miRNA pathway, namely that there are interactions between the mammalian FMRP and miRNAs and components of their pathway, such as Dicer and AGO2, which are all components of RISC [56, 58, 59]. Muddashetty et al. have shown that there is significantly reduced miR-125a localization to dendrites and synapses in the mice lacking FMRP, explaining this as the inability of AGO2 and miR-125a guided RISC to assemble on PSD-95 mRNA [3]. Moreover, in the presence of phosphorylated FMRP the miR-125a-guided RISC mediates the PSD-95 translation repression, whereas upon the FMRP dephosphorylation, induced by dihydroxyphenylglycine (DHPG), the PSD-95 translation is de-repressed. However, the molecular mechanisms by which this regulation is accomplished are not known.

Similar to the role of FMRP in mediating the miR-125a translational repressor function on PSD-95 mRNA, it has been shown that the phosphorylation status of FMRP is critical in the regulatory function of FMRP, with respect to the miR-196a-mediated repression of HOXB8 mRNA [59]. This study proposed that that FMRP directly interacts with AGO2, via binding to a specific binding pocket in the MID domain of the AGO2 and that phosphorylated FMRP recruits RISC to specific mRNA targets containing either G-quadruplexes or U rich sequences via its interactions with the RISC component AGO2 [59]. However, this simplified model fails to account how does FMRP discriminate for the RISC containing the correct miRNA targeting the mRNA to which FMRP is bound. Previous studies determined that there is selectivity in the association of mouse brain miRNAs with FMRP [60], and this cannot be explained simply by the recognition of AGO2 by the phosphorylated FMRP, as this RISC component is common to all miRNA-guided RISCs.

**Fig 8:**
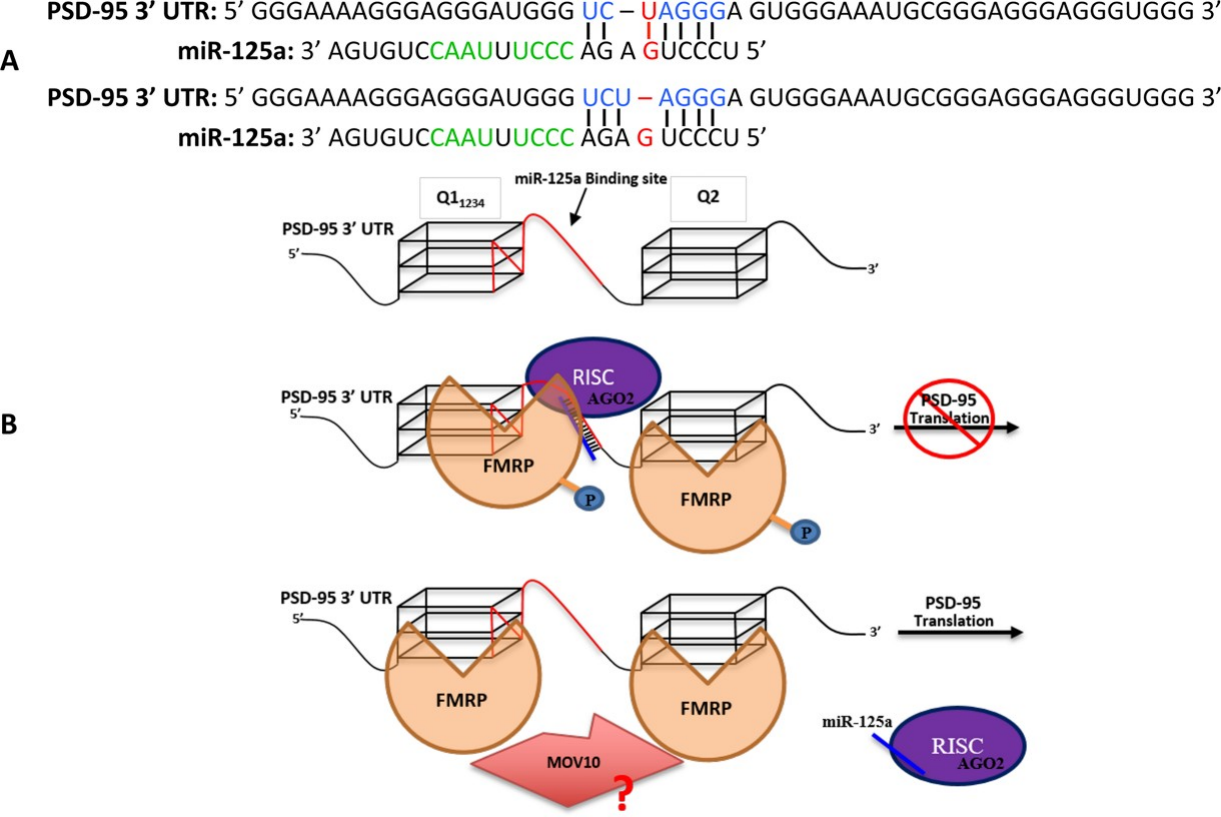
Proposed model for FMRP/ FMRP S500D in mediating the miR-125a translation regulation of PSD-95 mRNA. **(A)**. Predicted binding of miR-125a to PSD-95 mRNA. The PSD-95 mRNA sequence complementary to the miR-125a seed is highlighted in blue; the G-U base pair or the G-bulge predicted to form upon the miR-125a seed binding to its target onto PSD-95 mRNA is highlighted in red. The proposed sites onto miR-125a bound by the KH domains of FMRP S500D are shown in green. **(B).** Due to the presence of a weak binding between the miR-125a seed and its target sequence we postulate that FMRP S500D associates with miR-125a and potentially with Ago2 within RISC to confer additional interactions that stabilize the RISC-PSD-95 mRNA complex, inhibiting the PSD-95 mRNA translation. Upon the FMRP dephosphorylation induced by synaptic input, miR-125a and potentially AGO2 are no longer bound by FMRP, and the RISC-PSD-95 mRNA complex is no longer stable, resulting in the RISC dissociation. Potentially, the MOV10 helicase could also interact with FMRP to prevent the re-association of RISC.

Our novel findings that FMRP S500D binds the isolated miR-125a, in the absence of AGO2, whereas the unphosphorylated FMRP does not, could explain the selective recognition by the phosphorylated FMRP of the correct miR-125a guided RISC. Thus, we propose a refined model (Fig 8) according to which the phosphorylated FMRP facilitates the binding of the miR-125a-guided RISC to PSD-95 mRNA by using its KH1 and KH2 domains to recognize the CCCU nt 10–13 and UAAC nt 15–18 sites within miR-125a, and potentially also binding to AGO2 [59, 61], as well as by using its RGG box to bind the Q1 and Q2 G-quadruplexes. The model does not distinguish if the phosphorylated FMRP binds first PSD-95 mRNA via G-quadruplex recognition and then recruits miR-125a-RISC or if the pre-formed miR-125a-RISC-phosphorylated FMRP complex recognizes PSD-95 mRNA via miR-125a binding to its target site and FMRP binding to the G-quadruplex structures. We propose that the role of the phosphorylated FMRP in this context is to allow for the formation of a stable complex between the miR-125a-guided RISC and its target PSD-95 mRNA. The miR-125a seed does not have a perfectly complementary target sequence on PSD-95 mRNA, as it could either form a GU base pair (Fig 8A, top) or contain a bulged G (Fig 8A, bottom). Note that although the guide miR-125a nt at position 1 (g1), U, is complementary to the corresponding target position 1 (t1), A, on PSD-95 mRNA, this base pair does not form in the presence of AGO2. Crystal structures of AGO2, bound to miRNA and to the miRNA-target duplex showed that g1, which is in many cases U, is buried within a pocked in the AGO2 MID domain, unavailable for binding [62], whereas t1 on the target, which is in many cases A, inserts into a narrow pocket formed between the AGO2 MID and L2 domains [62].

It has been shown that miRNA seed sequences forming a single GU base pair with the mRNA target can be functional *in vivo* in the context of a 7-mer seed, however their translation repression efficiency is low [63]. For example, a GU wobble base pair in the context of a miRNA seed-target pairing is detrimental to the miRNA translation repression, reducing its efficiency by 16 fold, despite the fact that this pairing was not predicted to reduce the stability of the miRNA: mRNA interaction [64]. A series of crystal structures of the miRNA-loaded AGO2 bound to an 11 nt mRNA target containing complementary sequences to the miRNA nucleotides g2-g7 (6-mer seed), g2-g8 (7-mer seed) and g2-g9 (8-mer seed) [62] revealed that the primary mode of interaction of AGO2 is through the shape complementary recognition of the minor groove formed by the miRNA seed-target RNA duplex. Extensive hydrophobic and van der Waals contacts were observed between AGO2 and nts 2 to 7 of the miRNA-target RNA duplex [58], potentially explaining why the GU wobble base pair within nts 2 to 7 of the miRNA-target RNA duplex is so detrimental to translation repression efficiency, as this base pair structurally perturbs the minor groove of a canonical A type helix [65], reducing the AGO2 binding affinity.

The translation repression function of miRNA seed-target pairings containing bulged nucleotides in either the seed or the mRNA is also negatively impacted, only some of such miRNAs being functional *in vivo*, especially those supported by extensive complementarity between the miRNA 3’-end and the mRNA target [66]. It has been shown that single or double nucleotide seed mismatches have a negative impact reducing the k_on_ of the RISC binding to an mRNA target by 6–10 fold, especially when they occur at positions g2-g6 [62]. However, seed mismatches affect most drastically the k_off_ rate, as RISC has been shown to dissociate from mismatched targets 70–3200 faster than from perfectly matched seed-targets, again with mismatches at g2-g6 positions having the most impact [66]. While 3’ supplemental base pairing beyond the seed sequence does not increase the stability of the RISC-target complex in the case of a perfectly matched 7-mer strong seed, in the case of a weaker AU-rich seed the additional 3’ supplemental base pairing slowed more than 7-fold the RISC dissociation [66]. This suggests that although the mRNA target recognition is primarily dictated by the seed recognition, in the case of weaker or 1–2 nt mismatched seeds, complementarity to the center and 3’ end of the miRNA could compensate, resulting in the formation of a stable RISC-mRNA target complex [66].

The comparison of the crystal structures of AGO2-guide and AGO2-guide-target duplex [62] reveals rearrangements in both, the protein and the miRNA, organizing the guide nts g11-g16 to adopt an almost A type conformation with their Watson-Crick faces exposed to the solvent, able to form additional base pairs with the target RNA, thus contributing to the stability of the miRNA-target mRNA complex. The miRNA guide nts 17–19 appear to be conformationally heterogeneous, their role in formation of supplemental miRNA-mRNA base pairs being unclear.

In the case of miR-125a, the formation of supplemental base pairs beyond the seed sequence with PSD-95 mRNA is not possible, since the complementary bases for the guide past nt 8 are buried within the Q1 G-quadruplex (Fig 1), and thus not available for binding. Given that miR-125a does not have a perfectly matched 7-mer seed, forming either a GU wobble or a bulged G in the seed-target pairing (Fig 8A), and that it cannot form any 3’ supplemental base pairs with PSD-95 mRNA, we predict that in the absence of other factors, it will not form a stable RISC-PSD-95 mRNA complex, likely due to an increased dissociation rate k_off_. This prediction is supported by our findings that when the PSD-95 Q1 G-quadruplex is folded (in the presence of KCl), preventing any 3’ supplemental base pairing beyond the seed between miR-125a and its target, only a small fraction of miR-125a is stably complexed with PSD-95 Q1-Q2 mRNA (Fig 7A). In contrast, when the PSD-95 Q1 G-quadruplex is unfolded (in the presence of LiCl) and the entire miR-125a binding site is exposed, miR-125a forms a stable complex with PSD-95 Q1-Q2 mRNA (Fig 7B) at nM concentrations. We propose that the role of phosphorylated FMRP is to increase the stability of the miR-125a-RISC-PSD-95 mRNA complex by binding to the miR-125a nt 10–13 and 15–18 and potentially to AGO2 [58] thus, substituting for the miRNA-mRNA 3’ supplemental base pairing (Fig 8B, middle). When FMRP is dephosphorylated in response to synaptic input, it can no longer bind to miR-125a (Fig 5A and S4 Fig) and AGO2 [62], leading to the destabilization of the miR-125a-RISC-PSD-95 mRNA complex and resulting in the translation de-repression of PSD-95 mRNA (Fig 8B bottom). The helicase MOV10 has been shown to also bind G-quadruplex structures and when bound to G-rich sequences in the presence of FMRP to prevent the RISC association [67]. Thus, MOV10 might interact with the unphosphorylated FMRP to prevent the re-association of RISC. According to our model, the formation of a functional miR-125a-guided RISC complex onto PSD-95 mRNA requires the presence of phosphorylated FMRP. While our data does not provide *direct* unambiguous evidence for the above model, our EMSA binding experiments showing that when FMRP (unphosphorylated or S500D) was digested by proteinase K, following its incubation with miR-125a and PSD-95 Q1-Q2 mRNA, the same fraction of miR-125a was complexed with PSD-95 Q1-Q2 mRNA (Fig 7A), demonstrate that FMRP does not have nucleic acid chaperone activity with respect to the miR-125a-PSD-95 Q1-Q2 complex. Thus, in the case of this miRNA which has a weak seed and does not have the possibility of forming 3’ supplemental base pairs beyond the seed with its mRNA target, additional interactions are required to stabilize the complex. Such additional interactions are no longer required when supplemental interactions between miR-125a and PSD-95 Q1-Q2 are possible, as a stable miR-125a-PSD-95 Q1-Q2 complex is formed in the presence of LiCl when the Q1 G-Quadruplex is unfolded (Fig 7B).

Such an elegant design has the advantage that the miR-125a-mediated PSD-95 mRNA translation regulation is reversibly being turned on/off by using as a switch the dephosphorylated/ phosphorylated FMRP. While our experimental data has does not *directly* prove the mechanistic model trying to explain the role of FMRP in facilitating the miR-125a translation regulation of PSD-95 mRNA, this work should spark further research to test this model and moreover, to determine if such a switch involving the interactions of phosphorylated FMRP with miRNA to increase the stability of the RISC-mRNA target is unique to miR-125a or if it also exists for other miRNAs whose translation regulation function is mediated by FMRP.

## Supporting information

S1 Fig Denaturing Gel for PSD-95 samples. 20% denaturing gel for PSD-95 Q2, PSD-95 Q1_1234_, PSD-95 Q1 and PSD-95 Q1-Q2, showing that the sequences are pure and migrate according to their size differences.
(TIFF)

S2 Fig PSD-95 Q1-Q2 thermal denaturation experiment in 150 mM NaCl. These conditions were mimicking the preparation of the PSD-95 probe used in the pulldown experiment for Fig 4. PSD-95 Q1-Q2 was annealed in 150 mM KCl at 95°C for 5 minutes then equilibrated on the benchtop for 10 minutes. The prepared RNA was then diluted to 15 μM in the presence of 150 mM NaCl.
(TIFF)

S3 Fig miRNA-196a-1 binding with FMRP ISO1 and FMRP S500D. EMSA (15% non-denaturing gel) of miR-196a-1 with FMRP ISO1 **(left)** and FMRP S500D **(right)**. Free 200 nM miR-196a-1 was incubated with a 1:1 and 1:2 RNA: protein ratio. The samples were incubated with FMRP ISO1/FMRP S500D for 15 minutes at room temperature. The gel was visualized by staining with Syber Gold.
(TIFF)

S4 Fig FMRP binding to miR-125a at increasing ratios. EMSA (15% non-denaturing gel) of miR-125awith FMRP ISO1. 200 nM miR-125a was incubated with FMRP ISO1 on the bench for 15 minutes. The gel was visualized by staining with Syber Gold.
(TIFF)

S5 Fig PSD-95 mRNA thermal denaturation in LiCl. 10 μM PSD-95 Q1-Q2 mRNA was boiled for 5 minutes in the presence of 25 mM LiCl and cooled at room temperature for 10 minutes. The thermal denaturation experiment was performed at 295 nm to observe the hypochromic transition associated with the G-quadruplex dissociation. A single hypochromic transition is present, indicating that in LiCl the Q1 G-quadruplex is not stable (Stefanovic et al., 2015).
(TIFF)

S6 Fig The full unaltered gel images used to create Fig 2. (**A**) Binding of PSD-95 Q1 and Q1_1234_ to FMRP RGG. (**B**) Binding of PSD-95 Q2 to FMRP RGG. (**C**) Binding of PSD-95 Q1-Q2 to FMRP RGG. 200 nM RNA was incubated with FMRP on the bench for 15 minutes. 15% native (non-denaturing) gel electrophoresis was run and visualized by staining with Syber Gold.
(TIFF)

S7 Fig The full unaltered gel images used to create Fig 5. (**A**) miRNA-125a binding FMRP ISO1. (**B**) miRNA-125a, miRNA-125b, miRNA-125a-mut binding FMRP S500D. (**C**) miRNA-125a-mut2 binding FMRP S500D (lanes 4–7 from an unrelated experiment). (**D**) miRNA-122 binding FMRP / FMRP S500D. (**E**) miR-125a binding FMRP RGG (lanes 1–3, remaining lanes were control lanes. 200 nM RNA was incubated with FMRP on the bench for 15 minutes. 15% native (non-denaturing) gel electrophoresis was run and visualized by staining with Syber Gold.
(TIFF)

S8 Fig The full unaltered gel images used to create Fig 7. (**A**) Binding experiment performed in KCl. (**B**) Binding Experiment performed in LiCl.
(TIFF)
