# Supplementary figures and images for: FMRP - G-quadruplex mRNA - miR-125a interactions: Implications for miR-125a mediated translation regulation of PSD-95 mRNA
Source: PLoS One. 2019 May 21;14(5):e0217275. doi: 10.1371/journal.pone.0217275 (PMC6529005; doi:10.1371/journal.pone.0217275)

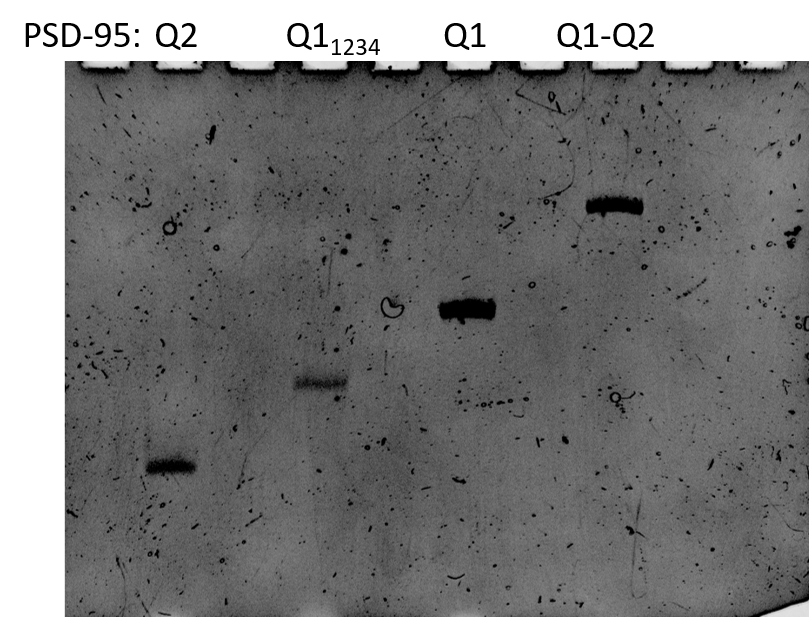

Supplement: S1 Fig — 20% denaturing gel for PSD-95 Q2, PSD-95 Q11234, PSD-95 Q1 and PSD-95 Q1-Q2, showing that the sequences are pure and migrate according to their size differences. (TIFF) [file pone.0217275.s001.tiff]

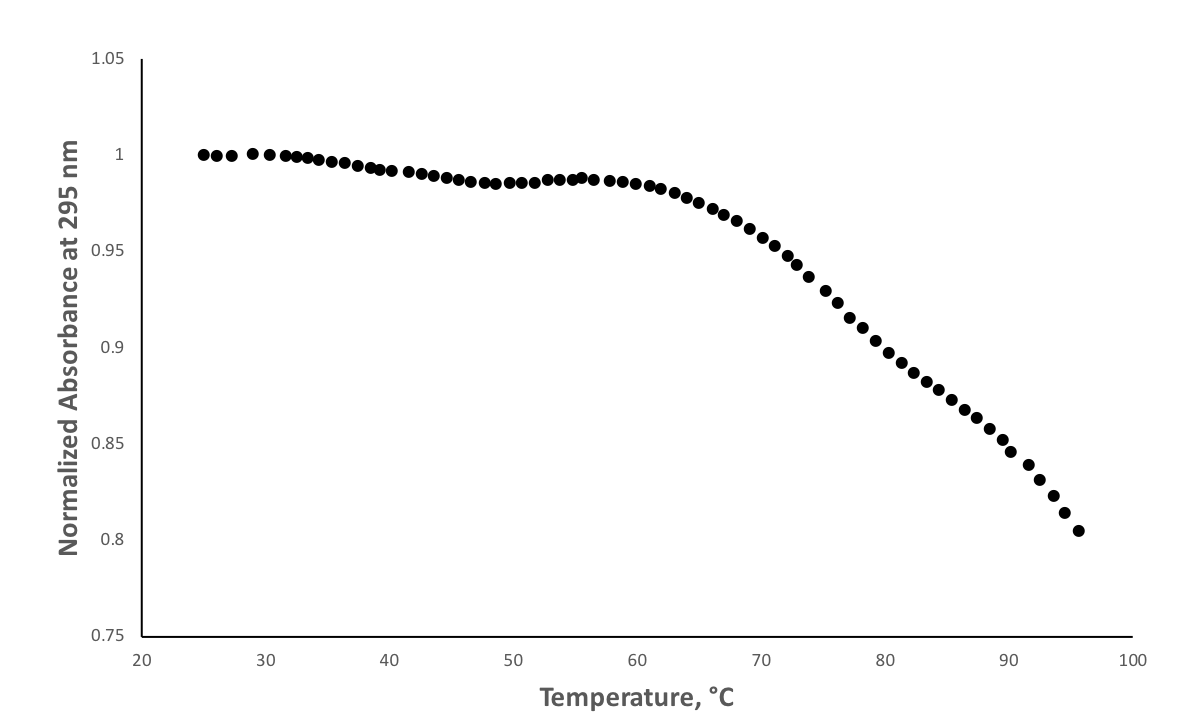

Supplement: S2 Fig — These conditions were mimicking the preparation of the PSD-95 probe used in the pulldown experiment for Fig 4. PSD-95 Q1-Q2 was annealed in 150 mM KCl at 95°C for 5 minutes then equilibrated on the benchtop for 10 minutes. The prepared RNA was then diluted to 15 μM in the presence of 150 mM NaCl. (TIFF) [file pone.0217275.s002.tiff]

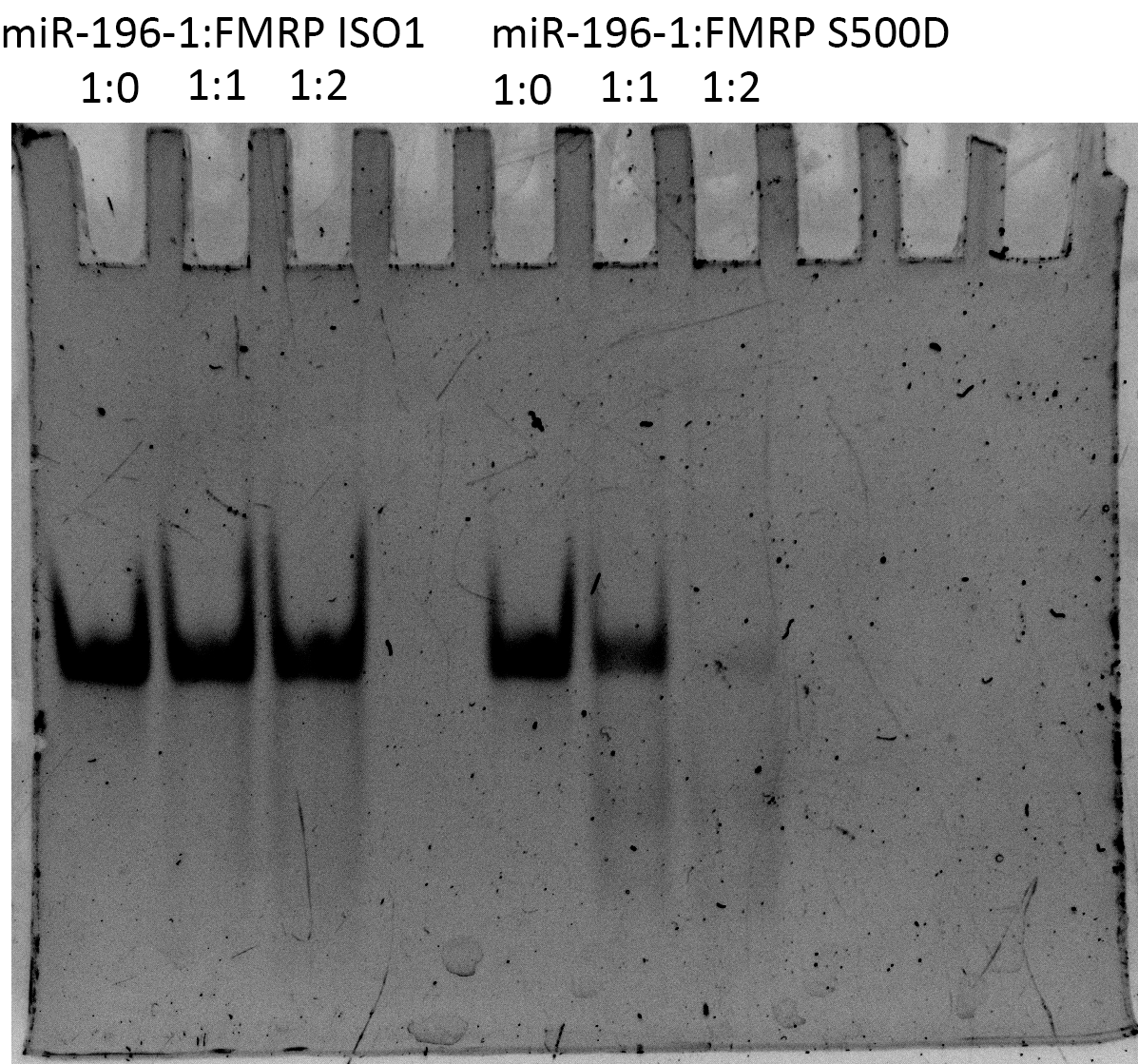

Supplement: S3 Fig — EMSA (15% non-denaturing gel) of miR-196a-1 with FMRP ISO1 (left) and FMRP S500D (right). Free 200 nM miR-196a-1 was incubated with a 1:1 and 1:2 RNA: protein ratio. The samples were incubated with FMRP ISO1/FMRP S500D for 15 minutes at room temperature. The gel was visualized by staining with Syber Gold. (TIFF) [file pone.0217275.s003.tiff]

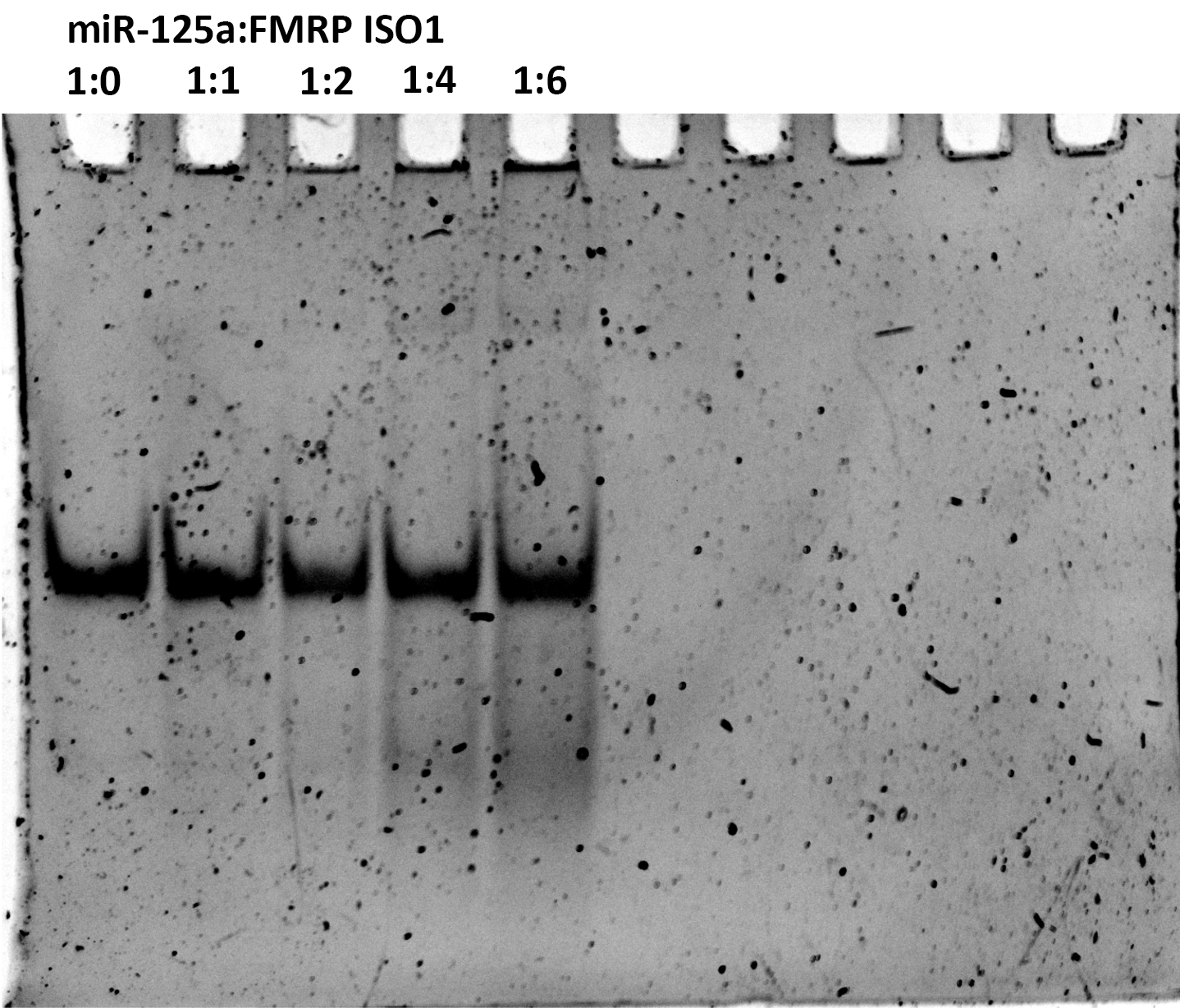

Supplement: S4 Fig — EMSA (15% non-denaturing gel) of miR-125awith FMRP ISO1. 200 nM miR-125a was incubated with FMRP ISO1 on the bench for 15 minutes. The gel was visualized by staining with Syber Gold. (TIFF) [file pone.0217275.s004.tiff]

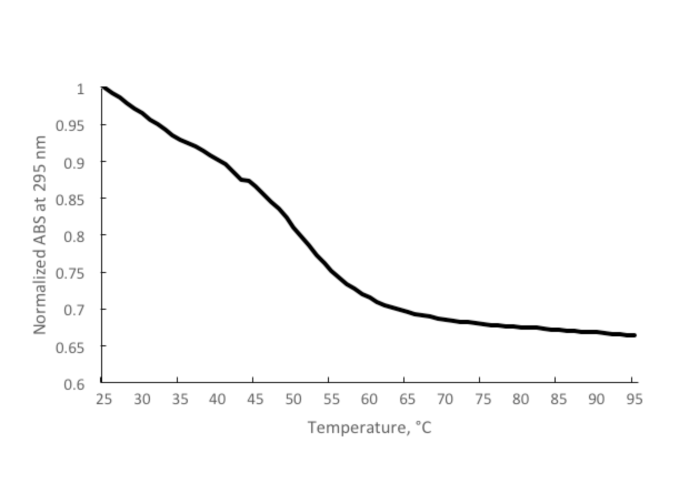

Supplement: S5 Fig — 10 μM PSD-95 Q1-Q2 mRNA was boiled for 5 minutes in the presence of 25 mM LiCl and cooled at room temperature for 10 minutes. The thermal denaturation experiment was performed at 295 nm to observe the hypochromic transition associated with the G-quadruplex dissociation. A single hypochromic transition is present, indicating that in LiCl the Q1 G-quadruplex is not stable (Stefanovic et al., 2015). (TIFF) [file pone.0217275.s005.tiff]

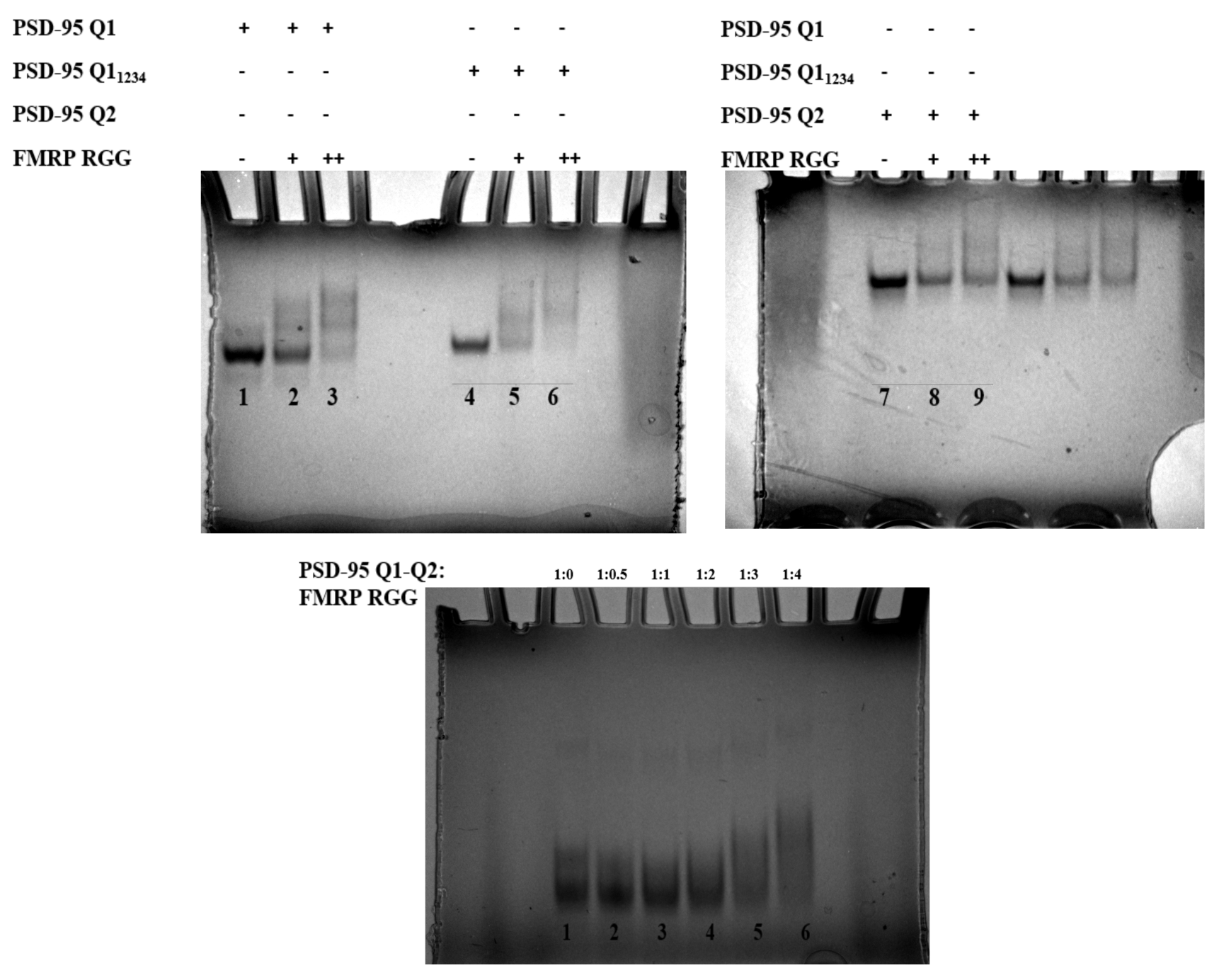

Supplement: S6 Fig — (A) Binding of PSD-95 Q1 and Q11234 to FMRP RGG. (B) Binding of PSD-95 Q2 to FMRP RGG. (C) Binding of PSD-95 Q1-Q2 to FMRP RGG. 200 nM RNA was incubated with FMRP on the bench for 15 minutes. 15% native (non-denaturing) gel electrophoresis was run and visualized by staining with Syber Gold. (TIFF) [file pone.0217275.s006.tiff]

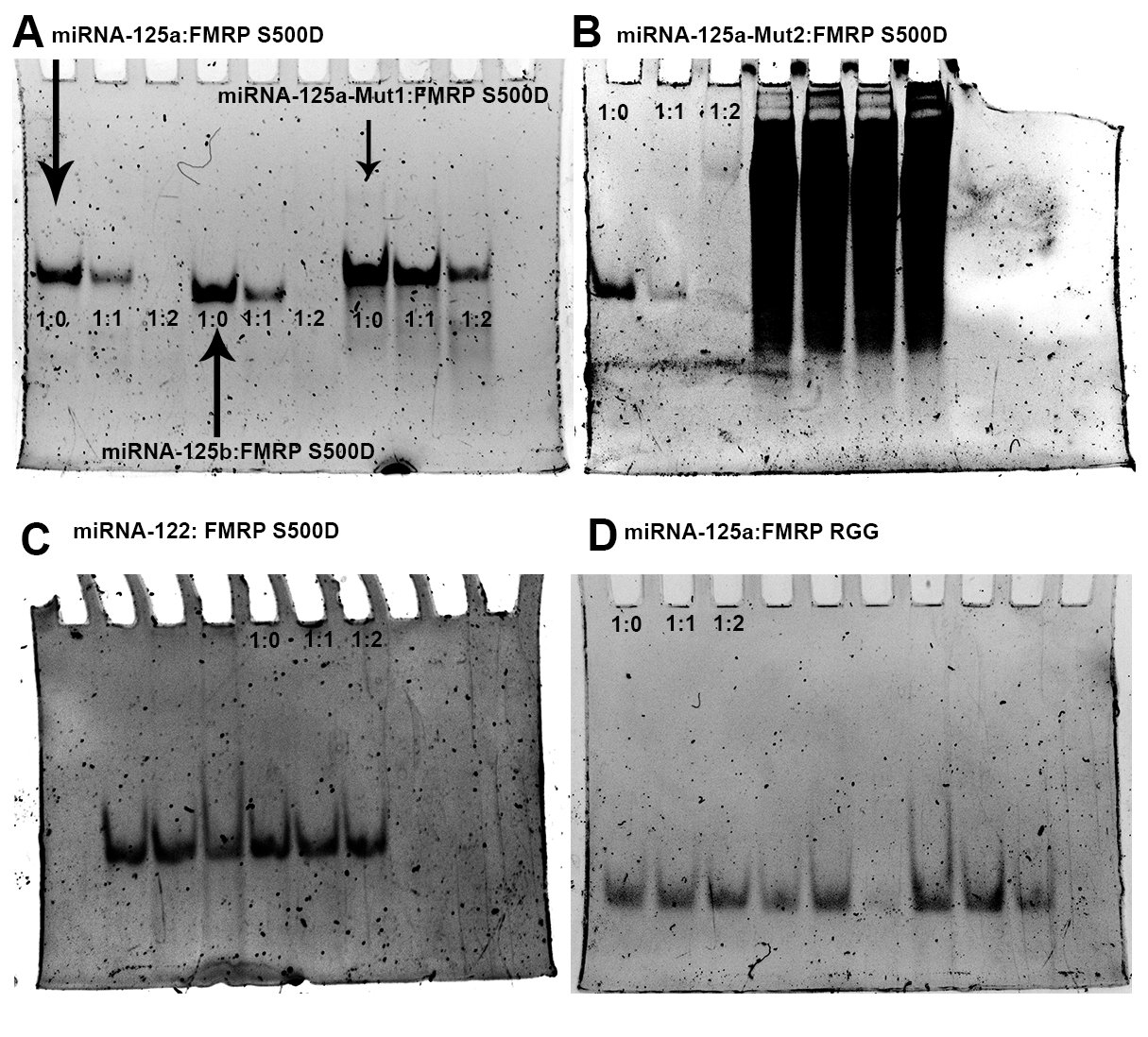

Supplement: S7 Fig — (A) miRNA-125a binding FMRP ISO1. (B) miRNA-125a, miRNA-125b, miRNA-125a-mut binding FMRP S500D. (C) miRNA-125a-mut2 binding FMRP S500D (lanes 4–7 from an unrelated experiment). (D) miRNA-122 binding FMRP / FMRP S500D. (E) miR-125a binding FMRP RGG (lanes 1–3, remaining lanes were control lanes. 200 nM RNA was incubated with FMRP on the bench for 15 minutes. 15% native (non-denaturing) gel electrophoresis was run and visualized by staining with Syber Gold. (TIFF) [file pone.0217275.s007.tiff]

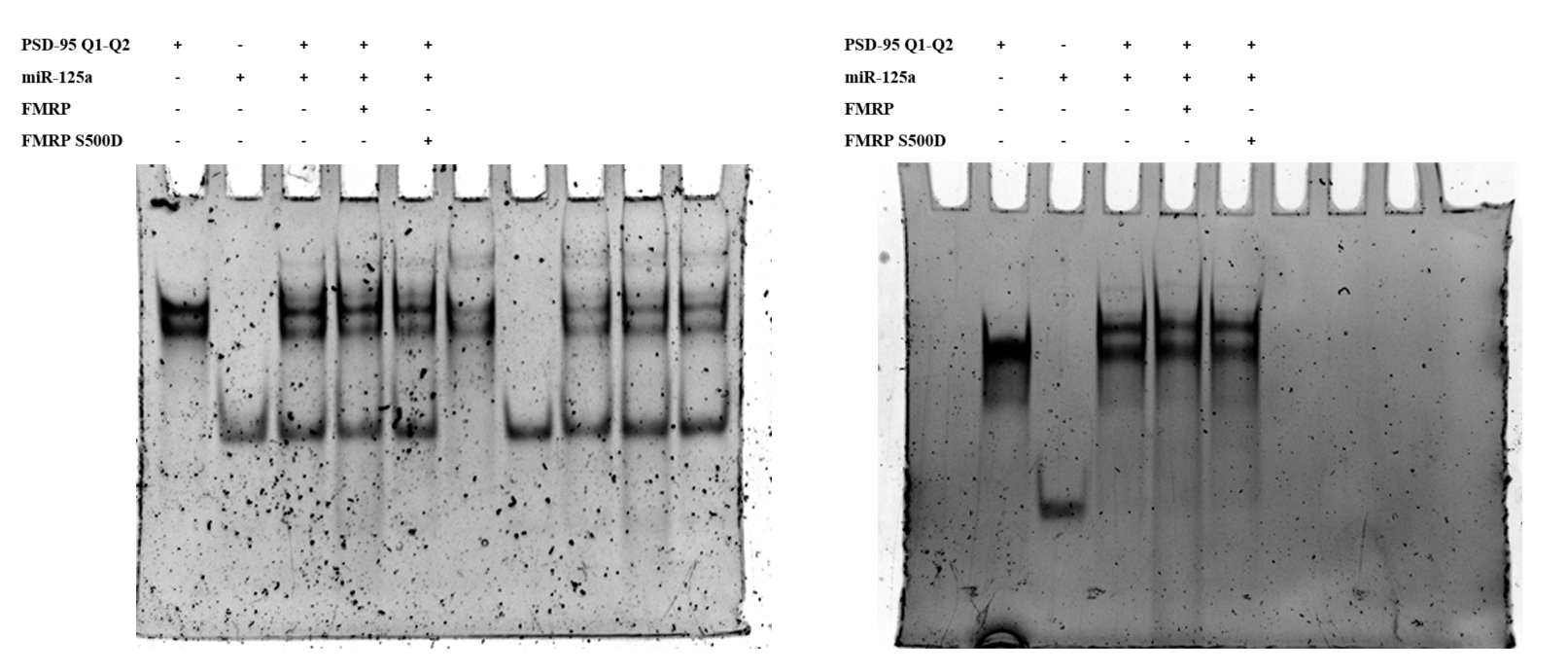

Supplement: S8 Fig — (A) Binding experiment performed in KCl. (B) Binding Experiment performed in LiCl. (TIFF) [file pone.0217275.s008.tiff]
